# Comprehensive Characterization and Global Transcriptome Analysis of Human Fetal Liver Terminal Erythropoiesis

**DOI:** 10.1016/j.gpb.2023.07.001

**Published:** 2023-08-30

**Authors:** Yongshuai Han, Shihui Wang, Yaomei Wang, Yumin Huang, Chengjie Gao, Xinhua Guo, Lixiang Chen, Huizhi Zhao, Xiuli An

**Affiliations:** 1 School of Life Sciences, Zhengzhou University, Zhengzhou 450001, China; 2 Laboratory of Membrane Biology, New York Blood Center, New York, NY 10065, USA; 3 Institute of Hematology, People’s Hospital of Zhengzhou University, Zhengzhou 450003, China; 4 Department of Hematology, the Affiliated Cancer Hospital of Zhengzhou University, Zhengzhou 450000, China; 5 Department of Hematology, the First Affiliated Hospital of Zhengzhou University, Zhengzhou 450002, China

**Keywords:** Human fetal liver, Terminal erythropoiesis, Transcriptome, Immortalized erythroid cell line, Enucleation

## Abstract

The fetal liver (FL) is the key erythropoietic organ during fetal development, but knowledge on human FL erythropoiesis is very limited. In this study, we sorted primary erythroblasts from FL cells and performed RNA sequencing (RNA-seq) analyses. We found that temporal gene expression patterns reflected changes in function during primary human FL **terminal erythropoiesis**. Notably, the expression of genes enriched in proteolysis and autophagy was up-regulated in orthochromatic erythroblasts (OrthoEs), suggesting the involvement of these pathways in **enucleation**. We also performed RNA-seq of *in vitro* cultured erythroblasts derived from FL CD34^+^ cells. Comparison of **transcriptomes** between the primary and cultured erythroblasts revealed significant differences, indicating impacts of the culture system on gene expression. Notably, the expression of lipid metabolism-related genes was increased in cultured erythroblasts. We further **immortalized erythroid cell lines** from FL and cord blood (CB) CD34^+^ cells (FL-iEry and CB-iEry, respectively). FL-iEry and CB-iEry were immortalized at the proerythroblast stage and can be induced to differentiate into OrthoEs, but their enucleation ability was very low. Comparison of the transcriptomes between OrthoEs with and without enucleation capability revealed the down-regulation of pathways involved in chromatin organization and mitophagy in OrthoEs without enucleation capacity, indicating that defects in chromatin organization and mitophagy contribute to the inability of OrthoEs to enucleate. Additionally, the expression of *HBE1*, *HBZ*, and *HBG2* was up-regulated in FL-iEry compared with CB-iEry, and such up-regulation was accompanied by down-regulated expression of *BCL11A* and up-regulated expression of *LIN28B* and *IGF2BP1*. Our study provides new insights into human FL erythropoiesis and rich resources for future studies.

## Introduction

Erythropoiesis is a process by which red blood cells are produced. It occurs first in the yolk sac as the “primitive” form, and is then gradually replaced by the “definitive” form in the fetal liver (FL) and bone marrow (BM) during fetal development and after birth [1], [2]. The earliest committed erythroid progenitor is the burst-forming unit-erythroid (BFU-E); the progenitors differentiate into a late-stage erythroid progenitor, the colony-forming unit-erythroid (CFU-E). CFU-Es undergo terminal erythroid differentiation to become erythroid precursors, including proerythroblasts (ProEs), basophilic erythroblasts (BasoEs), polychromatic erythroblasts (PolyEs), and orthochromatic erythroblasts (OrthoEs) [3], [4]. During terminal erythroid differentiation, cell size progressively decreases, while chromatin condensation and hemoglobin synthesis increase. Finally, OrthoEs expel their nuclei to become reticulocytes, which mature into red blood cells in the bloodstream [5]. These changes enable the morphologic differentiation of erythroblasts at different developmental stages.

For more than 10 years, flow cytometry-based methods using surface markers to separate both mouse and human erythroid cells at each developmental stage have been developed by us and others [3], [6], [7], [8], [9], [10]. These novel methods have enabled the study of normal and disordered erythropoiesis in a stage-specific manner [11], [12], [13], [14], [15], [16]. Global transcriptomic, proteomic, and epigenetic analyses of these cell populations have provided novel insights into the biology of erythropoiesis [17], [18], [19]. Our previous transcriptome analyses of human and murine erythroblasts revealed significant stage- and species-specific differences across stages of terminal erythroid differentiation [17]. The epigenetic landscape of human erythropoiesis reveals that erythroid cells exhibit chromatin accessibility patterns distinct from other cell types. It also reveals stage-specific patterns of regulation [19]. However, it should be noted that for human erythropoiesis, all omics analyses were performed on *in vitro* cultured erythroid cells derived from cord blood (CB) or peripheral blood (PB) CD34^+^ cells. In marked contrast, there is a lack of knowledge on human FL erythropoiesis.

In the present study, we first showed that the surface markers we identified for separating cultured human erythroblasts [3] could be used to sort primary erythroblasts at different developmental stages from human FL cells. We then differentiated human FL CD34^+^ cells into erythroid cells *in vitro* using a three-phase erythroid culture system and showed that the *in vitro* erythropoiesis profile of FL CD34^+^ cells was more similar to that of CB CD34^+^ cells than to that of PB CD34^+^ cells. We performed RNA sequencing (RNA-seq) analyses on erythroblasts at each developmental stage sorted from both human FL and *in vitro* cultured erythroblasts derived from human FL CD34^+^ cells. Comparison of the transcriptomes between human primary FL erythroblasts and cultured erythroblasts derived from human FL CD34^+^ cells revealed many differences in gene expression between the primary human FL erythroblasts and cultured erythroblasts, indicating the influence of the culture system on terminal erythropoiesis. We also established immortalized erythroid cell lines from human FL cells and CB CD34^+^ cells, which we termed FL-iEry and CB-iEry, respectively. Characterization and RNA-seq analyses revealed that both FL-iEry and CB-iEry were immortalized at the ProE stage and could be induced to undergo terminal erythroid differentiation. Interestingly, the expression levels of embryonic and γ-globin genes were up-regulated in FL-iEry compared with CB-iEry. Together, our findings provide novel insights into human FL erythropoiesis. The establishment of an FL-iEry cell line should facilitate studies in human FL erythropoiesis.

## Results

### Combination of surface markers enables separation of primary erythroblasts from human FL cells

Using a three-phase erythroid culture system, we developed a flow cytometry-based method for separating human erythroblasts at each distinct developmental stage using glycophorin A (GPA), band 3, and α4-integrin as surface markers [3], [20], [21], [22]. This method can be used to separate primary erythroblasts at different stages of terminal erythroid differentiation in human BM with high purity [3]. We examined whether the combination of these markers could separate primary erythroblasts in human FL. Figure S1A shows the representative expression profile of band 3 *vs.* α4-integrin of GPA^+^ cells. Based on the expression levels of band 3 and α4-integrin, five clusters were gated and sorted. Cytospin analyses of the sorted cells showed that they morphologically resembled ProE, early BasoE, late BasoE, PolyE, and OrthoE (Figure S1B). These results demonstrate that the combination of the surface markers GPA, band 3, and α4-integrin can be used to obtain primary human FL erythroblasts at each distinct stage using fluorescence-activated cell sorting (FACS).

### Overall transcriptome profiles of primary human FL erythroblasts confirm FACS-based separation

**Figure 1:**
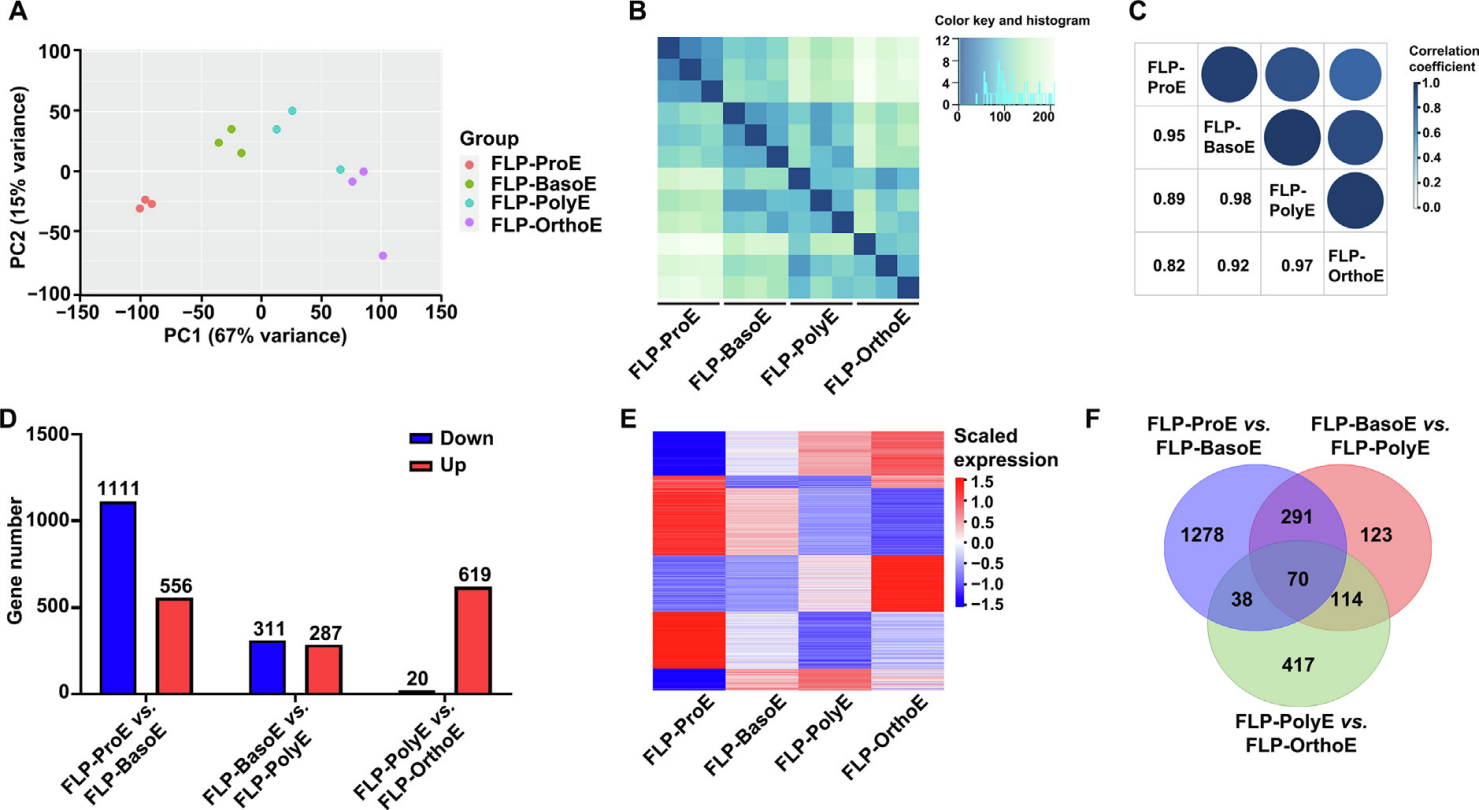
**RNA-seq analyses of primary erythroblasts from humanFL** **A.** PCA showing the separation of human FL primary erythroblasts at distinct developmental stages. **B.** Heatmap of pairwise distances between samples. **C.** Mixed plot of pairwise correlation coefficients between distinct stages of human FL primary erythroblasts. **D.** Bar plot of DEG numbers between adjacent stages. **E.** Heatmap representation of the gene expression of all DEGs from adjacent stages. **F.** Venn diagram of DEGs from adjacent stages. RNA-seq, RNA sequencing; FL, fetal liver; PCA, principal component analysis; DEG, differentially expressed gene; FLP-ProE, fetal liver primary proerythroblast; FLP-BasoE, fetal liver primary basophilic erythroblast; FLP-PolyE, fetal liver primary polychromatic erythroblast; FLP-OrthoE, fetal liver primary orthochromatic erythroblast.

To examine the changes in gene expression patterns during terminal differentiation of human FL cells *in vivo*, we obtained the transcriptome of the sorted primary human FL erythroblasts of each stage across terminal erythropoiesis. Consistent with our previous report [17], decreasing numbers of expressed genes were detected from ProE to OrthoE with 10,215, 9060, 7808, and 5750 expressed genes (≥ 1 transcript per million in at least one stage). All the expressed genes are listed in Table S1. Principal component analysis (PCA) showed clear separation of samples from each stage with the exception of one sample from PolyE (Figure 1A). Distance analysis revealed a shorter distance between samples within each stage and a longer distance between samples from different stages (Figure 1B). Pairwise correlation analyses revealed decreasing similarity in gene expression profiles during the differentiation process, from 0.95 between ProE and BasoE to 0.82 between ProE and OrthoE (Figure 1C).

We then compared gene expression between adjacent stages and obtained a total of 2331 differentially expressed genes (DEGs), with 1667 DEGs between ProE and BasoE, 598 DEGs between BasoE and PolyE, and 639 DEGs between PolyE and OrthoE (Figure 1D). A heatmap representation of all DEGs is shown in Figure 1E. A list of DEGs between adjacent stages is provided in Table S2. Notably, the greatest changes in gene expression (almost 70% of all DEGs) were seen between ProE and BasoE, indicating that the most notable changes occur at the early stage of human FL terminal erythropoiesis *in vivo*. A Venn diagram of DEGs between adjacent stages (ProE *vs.* BasoE, BasoE *vs.* PolyE, and PolyE *vs.* OrthoE) showed that 77% of DEGs identified in ProE *vs.* BasoE were found only in one comparison (Figure 1F), suggesting stage-specific changes from ProE to BasoE.

### Temporal patterns of gene expression during primary human FL terminal erythropoiesis reflect changes in function

**Figure 2:**
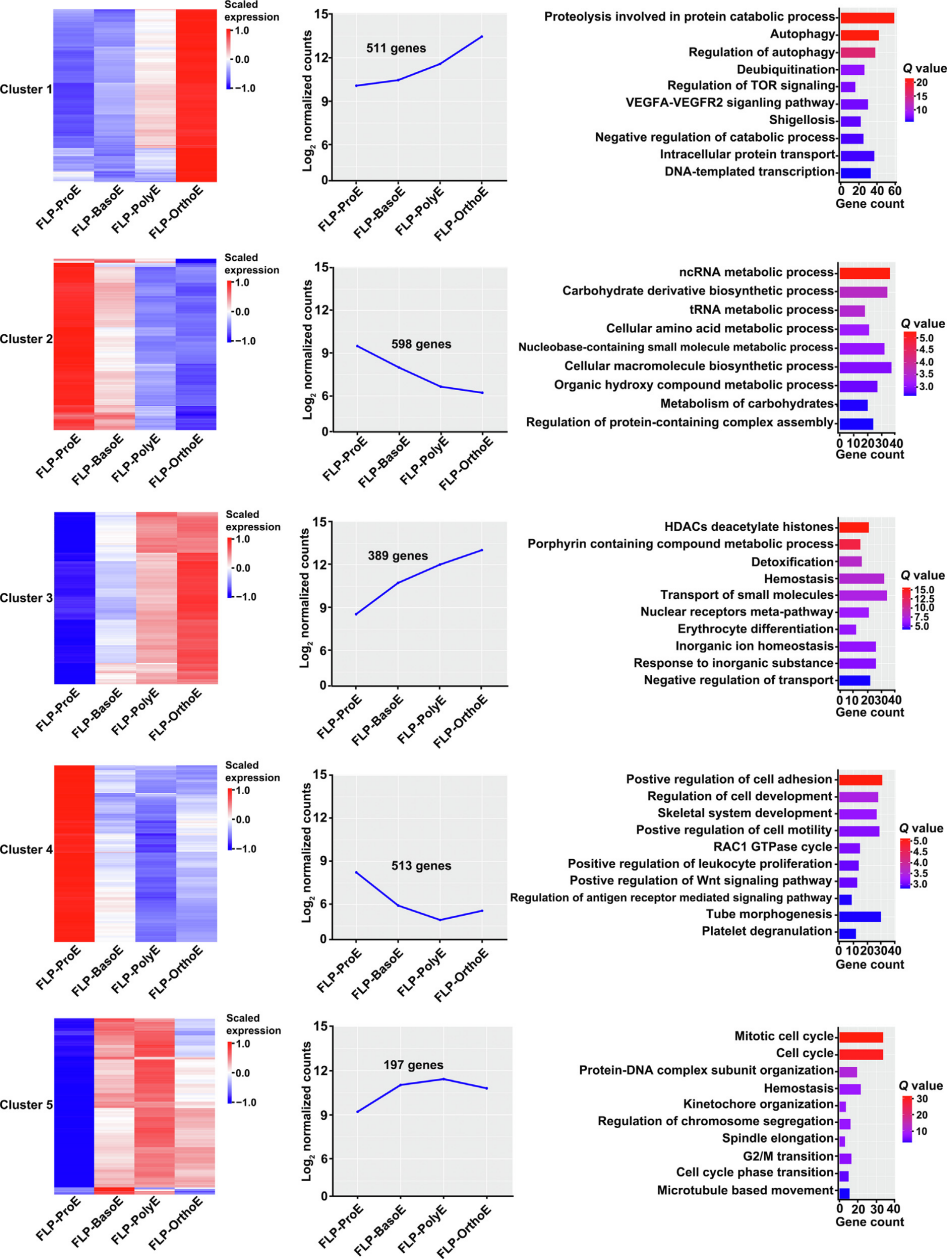
**Clusters of DEGs across stages during primary humanFL terminal erythropoiesis** The gene expression patterns of five identified clusters of all DEGs from adjacent-stage comparisons are shown in a heatmap (left panel) and a curve plot (middle panel). Enriched GO terms of genes within clusters are shown in a bar plot (right panel). The *Q* value represents the log-transformed adjusted *P* value. GO, Gene Ontology.

Dramatic changes occur during terminal erythropoiesis. To examine the dynamic changes in gene expression during this process, we performed a time-course analysis of the gene expression of all 2331 DEGs identified from the pairwise comparison of adjacent stages, followed by Gene Ontology (GO) enrichment analyses of the identified clusters. Heatmaps representing the gene expression patterns and their enriched GO terms are shown in Figure 2. The expression of genes in cluster 1 increased from ProE to OrthoE, with significant up-regulation in OrthoE. These genes were involved in proteolysis and autophagy, suggesting an increasing need for the clearance of proteins and organelles before enucleation. The genes in cluster 2, which showed decreasing expression from ProE to OrthoE, were enriched in the non-coding RNA (ncRNA) and transfer RNA (tRNA) metabolic processes, indicating a decrease in protein synthesis. The genes in cluster 3, which showed progressively increasing expression from ProE to OrthoE, were enriched in histone deacetylases, porphyrin-containing compound metabolic processes, and erythrocyte differentiation, consistent with increased hemoglobin synthesis and chromatin remodeling during terminal erythropoiesis. Gene expression in cluster 4 was characterized by abundant expression in ProE and decreasing expression at later stages. The genes in this group were enriched in positive regulation of cell adhesion, consistent with previous findings that adhesion molecules were decreased during human terminal erythropoiesis [3]. The expression of genes in cluster 5 was up-regulated from ProE to BasoE and then remained stable from BasoE to OrthoE. These genes were enriched in the cell cycle, implying a more active cell cycle in ProE than erythroblasts at later stages.

### The ***in vitro*** erythropoiesis profile of human FL CD34^+^ cells is more similar to that of CB CD34^+^ cells than that of PB CD34^+^ cells

**Figure 3:**
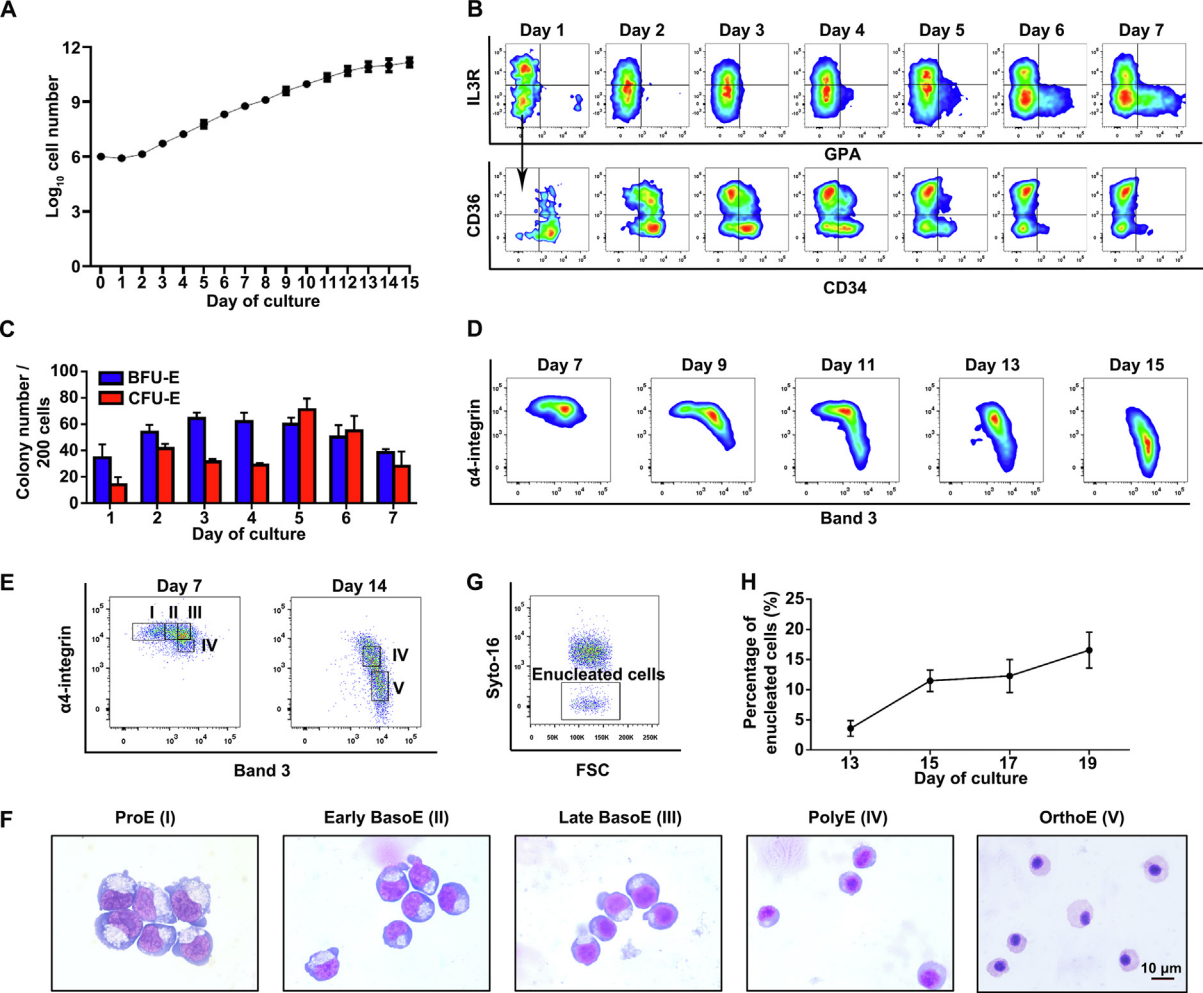
***In vitro* erythropoiesis profile of human FL CD34^+^ cells** **A.** Growth curve of the FL CD34^+^ cell-derived erythroid cells. Error bars indicate standard deviation (*n* = 6). **B.** Representative dot plots of GPA *vs.* IL-3R (upper panel) and CD34 *vs.* CD36 (lower panel) of GPA^−^IL-3R^−^ cells cultured from day 1 to day 7. **C.** Quantitation of colony-forming ability of BFU-E and CFU-E on the indicated days. Error bars indicate standard deviation (*n* = 3). **D.** Representative dot plots of band 3 *vs.* α4-integrin of GPA^+^ cells cultured from day 7 to day 15. **E.** Gating strategy of terminal erythroblasts on cultured day 7 or day 14 based on the expression levels of band 3 and α4-integrin of all GPA^+^ cells. Population I (ProE) was band3^neg^^ative^ α4-integrin^hi^^gh^, population II (early BasoE) was band3^low^ α4-integrin^hi^^gh^, population III (late BasoE) was band3^med^^ium^ α4-integrin^hi^^gh^, population IV (PolyE) was band3^med^^ium^ α4-integrin^med^^ium^, and population V (OrthoE) was band3^hi^^gh^ α4-integrin^low^. **F.** Representative images of sorted erythroblasts from the five populations shown in (E). Scale bar, 10 µm. **G.** Representative enucleation profile on cultured day 17. **H.** Quantitative analyses of enucleation. Error bars indicate the standard deviation (*n* = 3). BFU-E, burst-forming unit-erythroid; CFU-E, colony-forming unit-erythroid; GPA, glycophorin A.

Many previous studies used CB-, PB-, or BM-derived CD34^+^ cells to model neonatal or adult human erythropoiesis [23], [24], [25]. In the present study, we differentiated human FL CD34^+^ cells into the erythroid lineage using the three-phase erythroid culture system described in our previous studies [3], [20], [21], [22]. Figure 3A shows the growth curve of FL CD34^+^ cell-derived erythroid cells, showing that the cell population expanded approximately 263,000-fold over the 15-day culture period. We previously reported that BFU-E and CFU-E cells are immunophenotypically defined as GPA^−^IL-3R^−^CD34^+^CD36^−^ and GPA^−^IL-3R^−^CD34^−^CD36^+^, respectively [8]. Using this flow cytometry-based strategy, we monitored early-stage erythropoiesis during the first 7 days of culture. The representative expression profiles of GPA *vs.* IL-3R are shown in the upper panel of Figure 3B, which reveals a gradual acquisition of GPA accompanied by a decrease in IL-3R expression. The expression profiles of CD34 *vs.* CD36 are shown in the lower panel of Figure 3B, which reveals a gradual loss of CD34 expression accompanied by a gradual gain of CD36 expression. Yan et al. [10] showed that a subset of CD34^+^CD36^+^ cells, phenotypically located in the transition between BFU-E and CFU-E, were present with notable abundance in PB- but not CB-derived cultures from day 3 to day 6 of culture. Notably, FL-derived cultures presented a pattern similar to that of CB-derived cultures, indicating close similarity between FL and CB. We also examined the erythroid colony-forming ability of erythroid progenitors. Figure 3C shows that although BFU-E colonies peaked on day 3, CFU-E colonies peaked on day 5.

Next, we examined the terminal erythroid differentiation of FL CD34^+^ cells by flow cytometry using GPA, band 3, and α4-integrin as surface markers [3], [20], [26]. The expression profiles of band 3 *vs.* α4-integrin of GPA^+^ cells are shown in Figure 3D. As terminal erythroid differentiation proceeded, band 3 expression increased, whereas α4-integrin expression decreased. Based on the expression levels of band 3 and α4-integrin, we sorted ProE, early BasoE, and late BasoE from cells cultured for 7 days and sorted PolyE and OrthoE from cells cultured for 14 days. The gating strategies are shown in Figure 3E. Cytospin analyses of the sorted cells showed that the sorted cells morphologically resembled ProE, early BasoE, late BasoE, PolyE, and OrthoE, respectively (Figure 3F). These results indicate that the combination of GPA, band 3, and α4-integrin can be used to monitor terminal differentiation of FL CD34^+^ cells and isolate erythroblasts derived from FL CD34^+^ cells.

Enucleation is the last step of terminal erythroid differentiation. Therefore, we assessed enucleation using SYTO 16 staining by flow cytometry. The representative enucleation profiles and quantitative analyses are shown in Figure 3G and H, respectively. Unexpectedly, on day 17, the enucleation rate of erythroblasts derived from FL CD34^+^ cells only reached < 20%, which is significantly lower than that of erythroblasts derived from PB, CB, or BM CD34^+^ cells (the enucleation rate of which ranged from 45% to 65%) [20], [26], [27].

### Overall transcriptome profiles of cultured FL-derived erythroblasts reveal many more DEGs during ***in vitro*** terminal erythropoiesis

**Figure 4:**
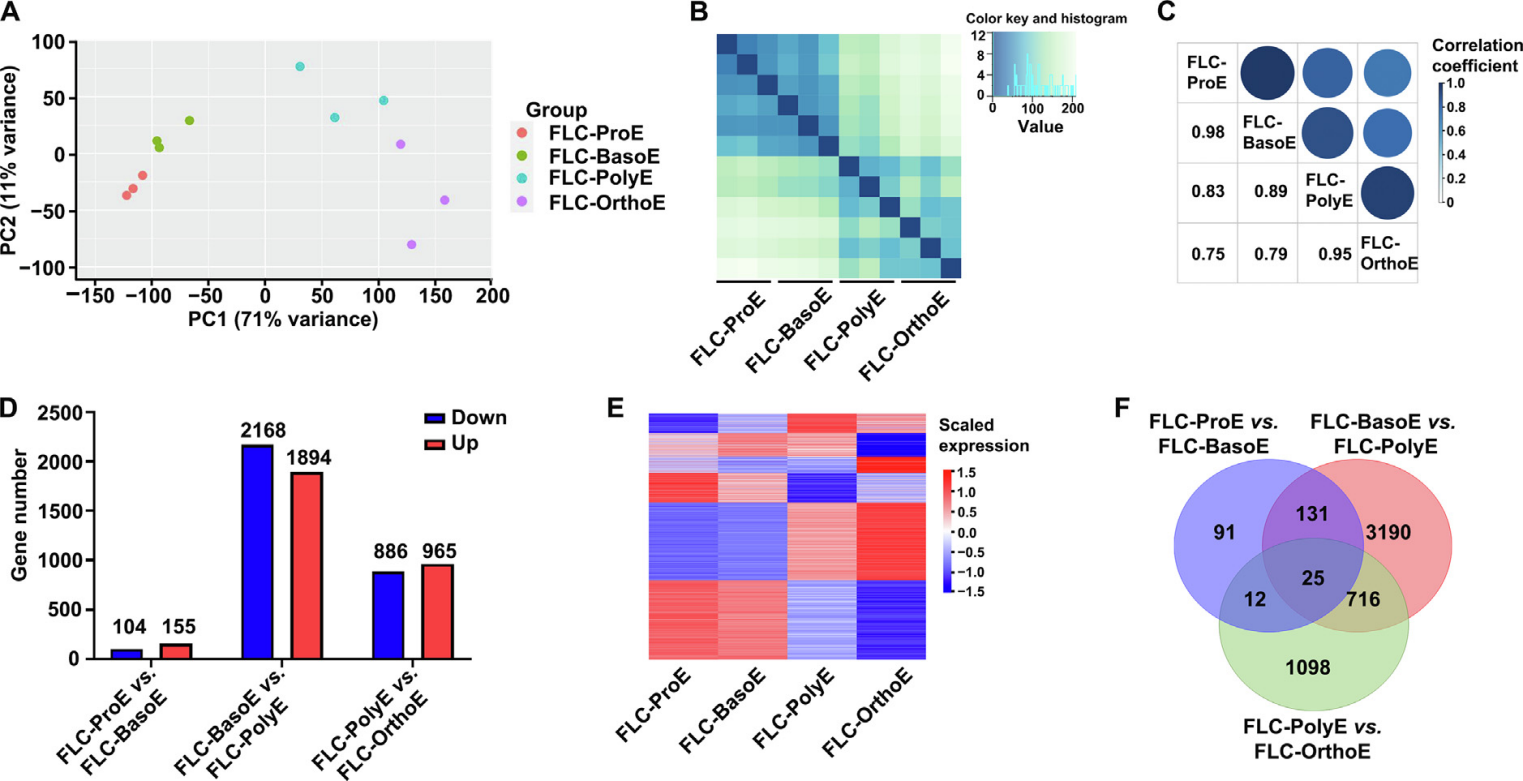
**RNA-seq analyses of cultured erythroblasts from human FL CD34^+^ cells** **A.** PCA showing the separation of FL cultured erythroblasts. **B.** Heatmap of pairwise distances between each stage of terminal erythroblasts. **C.** Mixed plot of pairwise correlation coefficients between stages. **D.** Bar plot of DEG numbers of adjacent stages. **E.** Heatmap representation of the gene expression of all DEGs from adjacent-stage comparisons. **F.** Venn diagram of DEGs from adjacent stages. FLC-ProE, fetal liver cultured proerythroblast; FLC-BasoE, fetal liver cultured basophilic erythroblast; FLC-PolyE, fetal liver cultured polychromatic erythroblast; FLC-OrthoE, fetal liver cultured orthochromatic erythroblast.

Next, we performed RNA-seq analyses of the cultured erythroblasts derived from human FL CD34^+^ cells. On average, the numbers of expressed genes were 10,115, 9620, 7240, and 5819 in the cultured ProEs, BasoEs, PolyEs, and OrthoEs, respectively, and these numbers were comparable to those of primary erythroblasts at the same stage. A list of expressed genes is provided in Table S3. PCA showed clear separation of the four stages (Figure 4A). We also analyzed the transcriptome profiles of primary and cultured erythroblasts using all expressed genes together and found different trajectories of *in vivo* and *in vitro* terminal erythropoiesis (Figure S2). The distance analysis showed that in contrast to the continuous separation of each stage of primary erythroblasts (Figure 1B), the cultured erythroblasts exhibited two-part separation, with ProE/BasoE as one part and PolyE/OrthoE as the other part (Figure 4B). Pairwise correlation analysis showed that the correlation coefficient between the cultured BasoEs and PolyEs was the smallest among all correlation coefficients between adjacent stages (Figure 4C), indicating more changes from BasoEs and PolyEs in the culture system. Pairwise transcriptome comparison between adjacent stages in cultured erythropoiesis revealed 5263 DEGs in total (Table S4), which was a much larger number than that in primary erythroblasts (2331 DEGs). Notably, of the total DEGs, 77% (4058 of 5263) were identified between the cultured BasoEs and PolyEs, whereas only 259 DEGs were identified between the cultured ProEs and BasoEs (Figure 4D). This indicated high similarity between the cultured ProEs and BasoEs but dramatic differences between the cultured BasoEs and PolyEs. A heatmap representing gene expression of all DEGs is shown in Figure 4E. Venn diagram analyses revealed that 83% of DEGs in cultured erythroblasts were only identified in one comparison (Figure 4F), indicating strong stage-specific changes from BasoEs to PolyEs. The many differences described above suggest different differentiation patterns of primary and cultured terminal erythropoiesis, most likely due to the effects of the *in vitro* culture environment.

### Predominant changes at PolyE and OrthoE stages during ***in vitro*** terminal erythropoiesis

**Figure 5:**
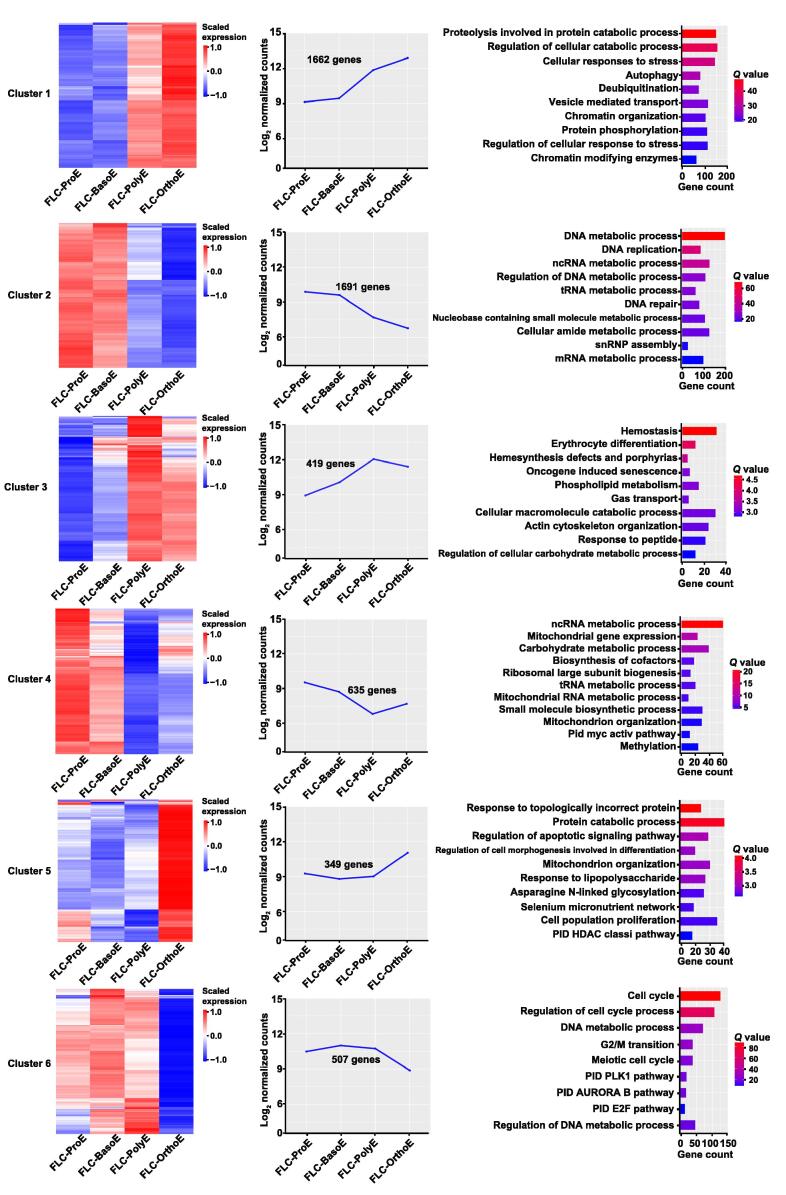
**Clusters of DEGs across stages during *in vitro* terminal erythropoiesis** The gene expression of six identified clusters of all DEGs from adjacent-stage comparisons in cultured terminal erythropoiesis is shown in a heatmap (left panel) and a curve plot (middle panel). Enriched GO terms of genes within clusters are shown in a bar plot (right panel). The *Q* value represents the log-transformed adjusted *P* value.

We next performed a time-course analysis of the expression patterns of all 5263 DEGs identified above to examine the dynamic patterns of gene expression during the *in vitro* terminal erythropoiesis of human FL CD34^+^ cells. As shown in Figure 5, six clusters were identified. Of these six clusters, only two were similar to the clusters of terminal erythropoiesis in primary human FL cells. In cluster 1, the expression of genes increased from ProE to OrthoE, and these genes were enriched in proteolysis and autophagy (similar to cluster 1 in primary erythropoiesis). In cluster 2, the expression of genes decreased from ProE to OrthoE, and these genes were enriched in the ncRNA, DNA, and tRNA metabolic processes (similar to cluster 2 in primary erythropoiesis). In cluster 3, the expression of genes increased from ProE to PolyE but slightly decreased in OrthoE, and these genes were enriched in hemostasis, erythrocyte differentiation, and phospholipid metabolism. In cluster 4, the expression of genes decreased from ProE to PolyE but slightly increased in OrthoE, and these genes were enriched in mitochondrial gene expression, mitochondrial RNA metabolic processes, and mitochondrion organization. In cluster 5, the expression of genes was relatively stable from ProE to PolyE and specifically up-regulated in OrthoE, and these genes were enriched in the cellular response to topologically incorrect proteins, regulation of the apoptotic signaling pathway, and mitochondrion organization. The expression of genes in cluster 6 was decreased and specifically down-regulated in OrthoE but enriched in portions of the cell cycle, such as G2/M transition and DNA metabolic processes. The patterns and GO enrichment of clusters 3–6 were not noted in human FL primary erythropoiesis but were similar to those reported in terminal erythropoiesis of CB CD34^+^ cells [17], demonstrating the similarity of erythroblasts cultured from CB CD34^+^ and FL CD34^+^ cells. The DEGs with up-regulated expression at each specific stage compared with other stages in FL *in vitro* and *in vivo* terminal erythropoiesis are listed in Table S5. Together, these results demonstrate that although there are conserved changes during *in vivo* and *in vitro* terminal erythropoiesis of human FL, *in vitro* culture leads to specific changes, particularly in the very late stage of terminal erythropoiesis.

### Increased lipid metabolism in cultured erythroblasts

**Figure 6:**
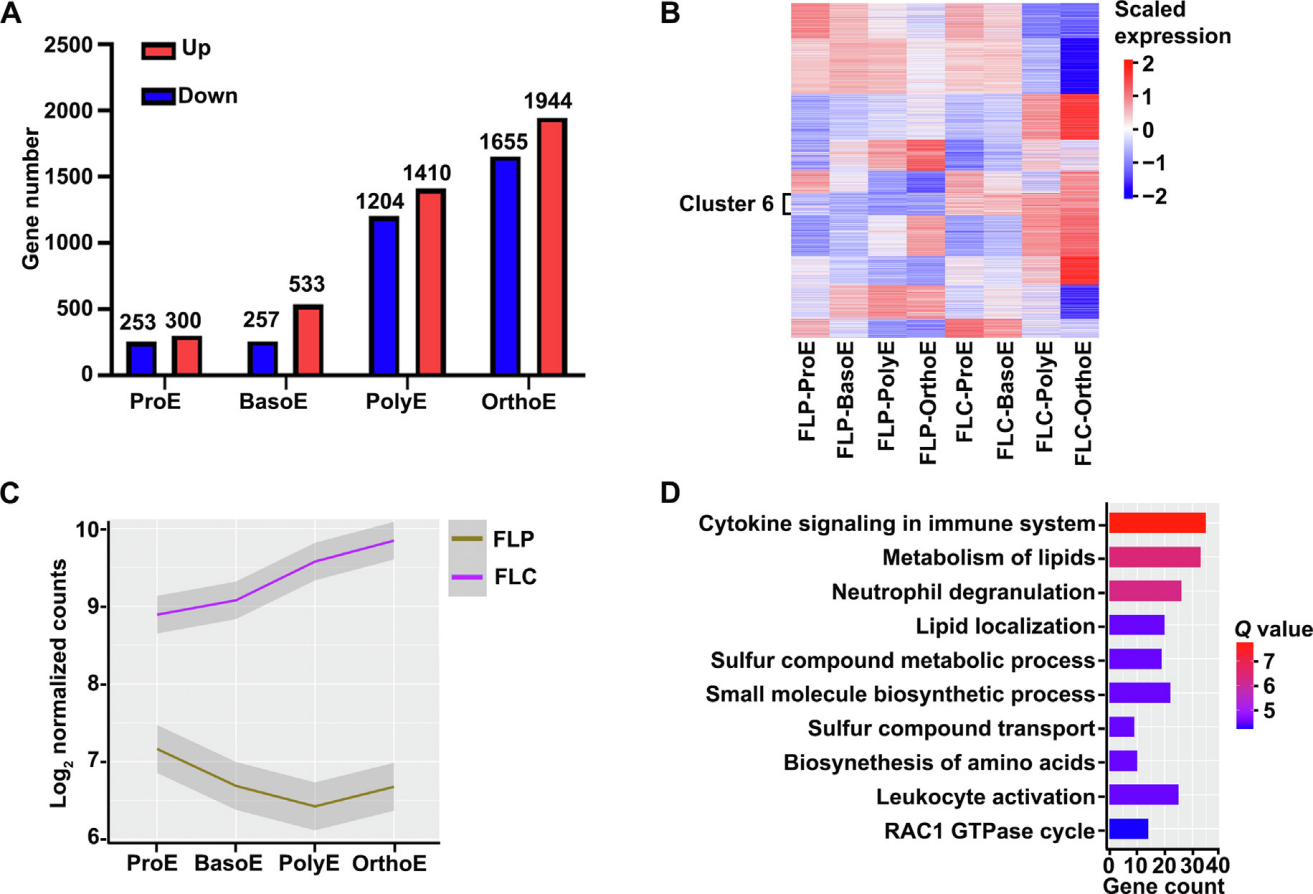
**Same-stage comparison between primary erythroblasts and cultured erythroblasts derived from human FL CD34^+^ cells** **A.** Bar plot of DEG numbers of same-stage comparison. **B.** Heatmap of the gene expression of all DEGs from the same-stage comparison. **C.** Curve representation of different expression levels of genes in cluster 6. **D.** Bar plot of enriched GO terms of genes in cluster 6. The *Q* value represents the log-transformed adjusted *P* value.

In the above-described results, we analyzed DEGs between adjacent stages during *in vivo* or *in vitro* terminal erythropoiesis of human FL. One notable limitation of such an approach is that it may fail to detect differences when the expression of a given gene does not change during terminal erythropoiesis. To clarify this issue, we next compared gene expression at the same developmental stage *in vivo* and *in vitro*. The numbers of DEGs between primary and cultured erythroblasts increased from ProE to OrthoE, with DEG numbers of 553, 790, 2614, and 3599 in ProE, BasoE, PolyE, and OrthoE, respectively. These results imply increased effects of the culture system on erythroblasts during the differentiation process (Figure 6A). A list of DEGs at each stage is provided in Table S6. We then performed clustering analyses of all 5148 DEGs identified above and identified 10 clusters (Figures 6B, Figure S3). Notably, among all these clusters, only one cluster (cluster 6) exhibited a stable expression pattern during both *in vitro* and *in vivo* terminal erythropoiesis. Interestingly, the expression levels of genes in this cluster were much higher in cultured erythroblasts than in primary erythroblasts at all stages (Figure 6C). These genes included genes involved in the metabolism of lipids (Figure 6D). Given that lipid metabolism is reportedly important in the energy production of terminal erythropoiesis [28], [29], these findings suggest that cultured erythroblasts may need higher energy supplies than primary erythroblasts.

### Generation and characterization of an immortalized erythroid cell line from human FL CD34^+^ cells

**Figure 7:**
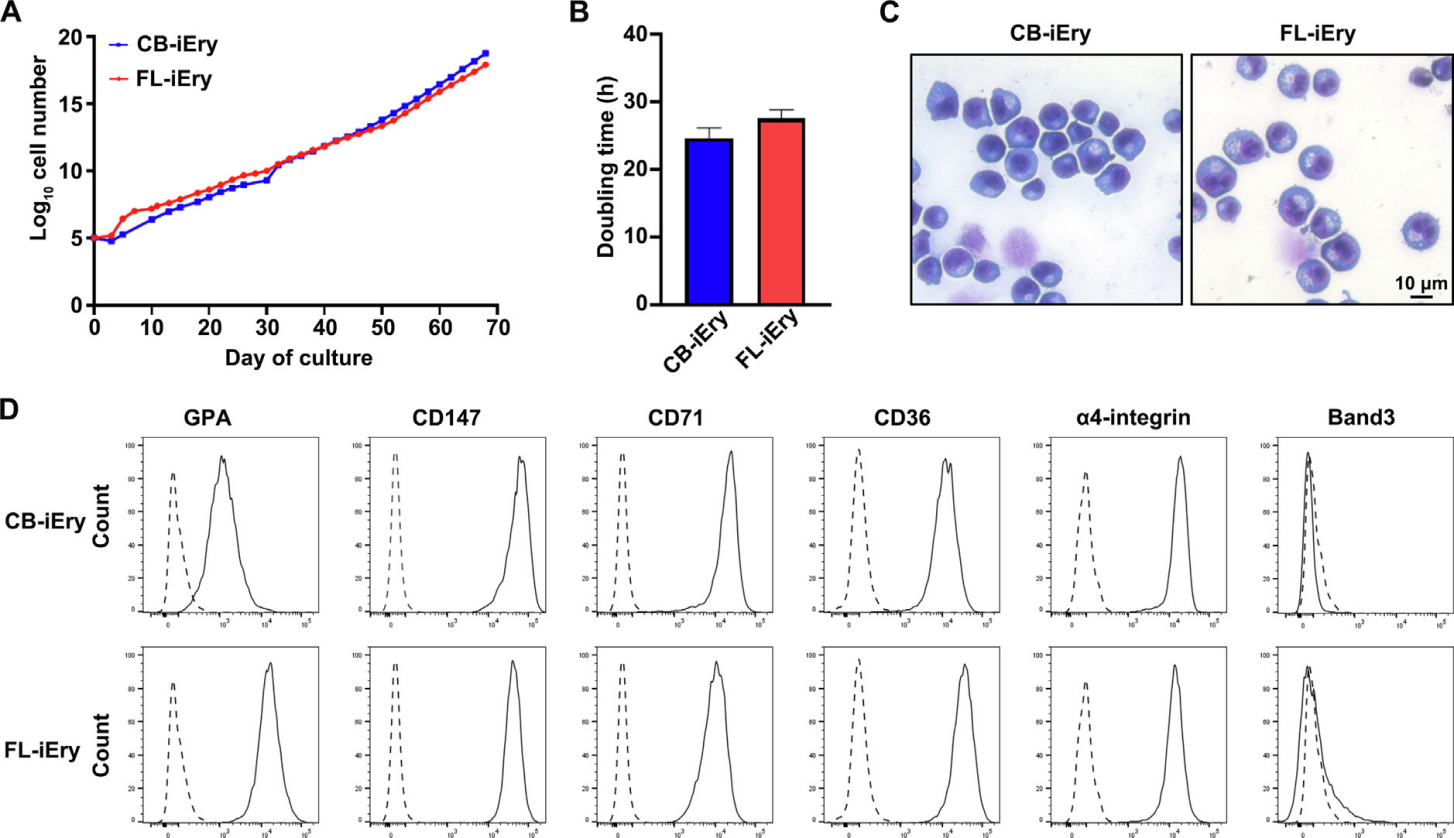
**Characterization of erythroid cell lines derived from FL and CB CD34^+^ cells** **A.** Growth curves of CB-iEry and FL-iEry in expansion medium. **B.** Doubling time determined by cell counting. **C.** Representative images of CB-iEry and FL-iEry. Scale bar, 10 µm. **D.** Flow cytometric analysis of the expression of membrane proteins at the cell surface of CB-iEry and FL-iEry. CB, cord blood; CB-iEry, cord blood CD34^+^ cell-derived immortalized erythroid cell; FL-iEry, fetal liver CD34^+^ cell-derived immortalized erythroid cell.

In other studies, immortalized erythroid cell lines have been generated from CB, PB, and BM CD34^+^ cells to model neonatal and adult human erythropoiesis [30], [31], [32]. However, an immortalized erythroid cell line from human FL is still lacking. To address this, we immortalized FL CD34^+^ cells under erythroid culture conditions utilizing a tetracycline-inducible human papillomavirus (HPV)-E6/E7 system [30], [31], [32]. As a control and for comparison purposes, we also immortalized CB CD34^+^ cells under the same conditions. Successful immortalization of the cell line was demonstrated by the fact that the cells continuously proliferated for more than 200 days. Figure 7A shows the growth curves of FL-iEry and CB-iEry with mean doubling times of 27 h and 24 h, respectively (Figure 7B). Cytospin analyses showed that the immortalized cells morphologically resembled early-stage erythroblasts (Figure 7C). We further examined the expression profile of surface membrane proteins by flow cytometry. As shown in Figure 7D, both FL-iEry and CB-iEry showed moderate expression of GPA; high expression of CD147, CD71, CD36, and α4-integrin; and no expression of band 3. Additionally, neither FL-iEry nor CB-iEry was able to form erythroid colonies (data not shown). Together, these results strongly suggest that the cell lines are immortalized at the ProE stage.

### Terminal erythroid differentiation profiles of FL-iEry and CB-iEry cell lines

**Figure 8:**
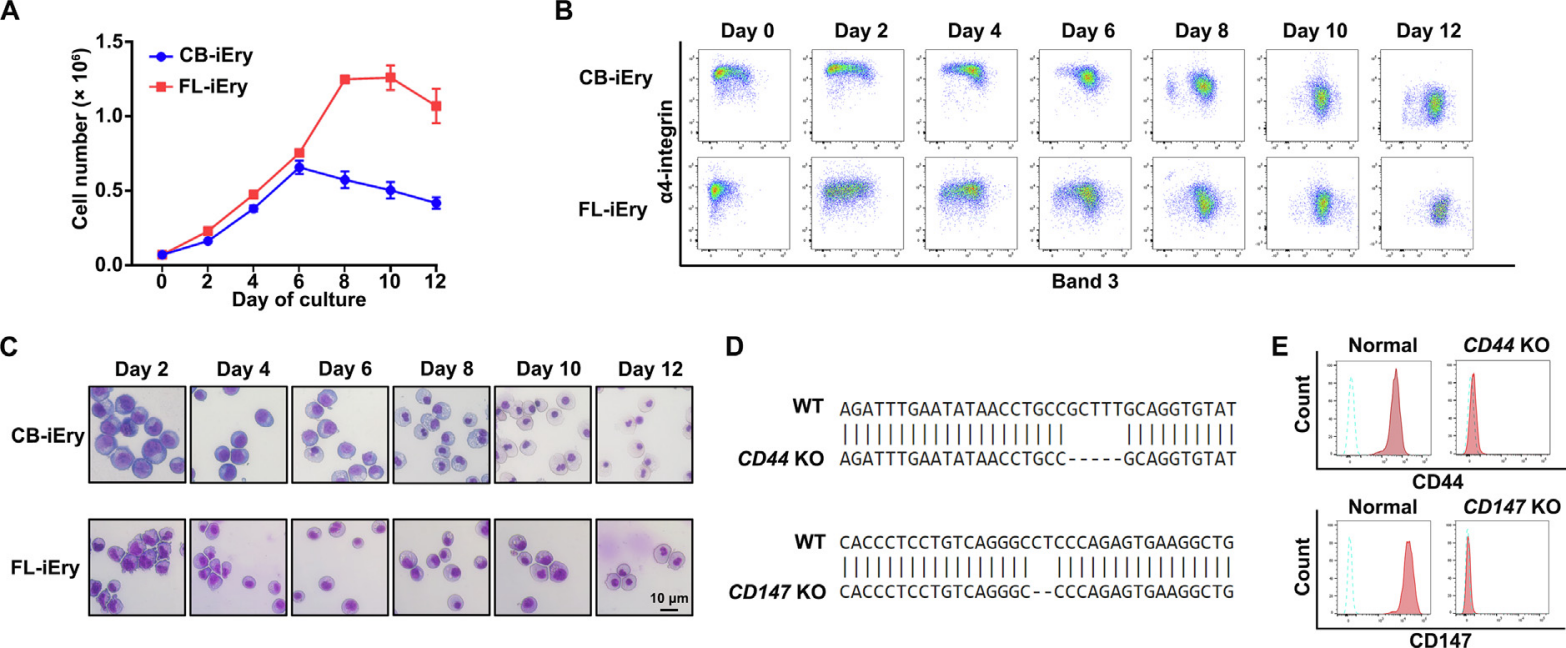
**Differentiation and gene knockout in FL and CB CD34^+^ cell-derived immortalized erythroid cells** **A.** Growth curves of CB-iEry and FL-iEry. **B.** Representative flow cytometry analysis of band 3 and α4-integrin expression. **C.** Representative cytospin image of erythroblasts from FL-iEry and CB-iEry on different differentiation days. **D.** Genome sequencing in *CD44* KO and *CD147* KO FL-iEry cell lines. **E.** Levels of CD44 and CD147 measured by flow cytometry after knockout of *CD44* or *CD147* in FL-iEry. KO, knockout.

To induce terminal erythroid differentiation, FL-iEry and CB-iEry cells maintained in expansion medium were transferred to differentiation medium. Figure 8A shows that in the differentiation medium, the proliferation of FL-iEry and CB-iEry cells lasted for 8 and 6 days, respectively, before they reached a plateau. Monitoring the terminal erythroid differentiation process by flow cytometry using band 3 and α4-integrin as markers showed that FL-iEry and CB-iEry underwent terminal erythroid differentiation in a similar manner, as demonstrated by the dynamic changes in the expression of band 3 and α4-integrin (Figure 8B). Cytospin analyses showed that morphologically, FL-iEry and CB-iEry progressively matured to BasoE, PolyE, and OrthoE, as demonstrated by a decrease in cell size and an increase in chromatin condensation (Figure 8C). Unexpectedly, the OrthoE cells derived from both FL-iEry and CB-iEry failed to enucleate (data not shown).

Immortalized human erythroid cell lines provide valuable tools for genetic manipulation to study human erythropoiesis *in vitro* [30], [31], [32]. We employed a CRISPR/Cas9 approach to delete two abundantly expressed blood group antigens, CD44 and CD147, in FL-iEry. Gene manipulation was confirmed by genome sequencing (Figure 8D), and deletion of protein expression was further confirmed by flow cytometry analyses (Figure 8E).

### Transcriptome analyses of immortalized erythroid cell lines reveal immortalization at the ProE stage

Next, we conducted RNA-seq analyses of the FL-iEry and CB-iEry cells and compared their transcriptomes with FL CD34^+^ cell-derived and CB CD34^+^ cell-derived erythroblasts at each developmental stage. PCA showed that CB-iEry and FL-iEry were closely clustered and separated from nonimmortalized erythroblasts and that CB-iEry and FL-iEry were closest to ProE (Figure S4A). This indicates that CB-iEry and FL-iEry are immortalized at the ProE stage, consistent with the morphology and flow cytometry analyses of the surface markers described above. Although the gene expression profiles of CB-iEry and FL-iEry were closest to ProE, differences were expected to exist between CB-iEry/FL-iEry and ProE. To examine these differences, we compared the transcriptomes of iEry and ProE from the same source: FL-iEry *vs.* FLC-ProE and CB-iEry *vs.* CB cultured proerythroblasts (CBC-ProE). In total, 1345 common DEGs were identified, and their expression levels are shown in Figure S4B and Table S7. Genes with up-regulated expression in iEry were enriched in meiotic synapses, including genes involved in telomere maintenance, such as *TERT* (Figure S4C and D). Given that overexpression of *TERT* leads to the generation of immortalized human cell lines [33], our findings suggest that establishment of the immortalized erythroid cell lines CB-iEry/FL-iEry by the HPV-E6/E7 element occurs at least in part via up-regulated expression of *TERT*.

### Differential hemoglobin expression between FL-iEry and CB-iEry

**Figure 9:**
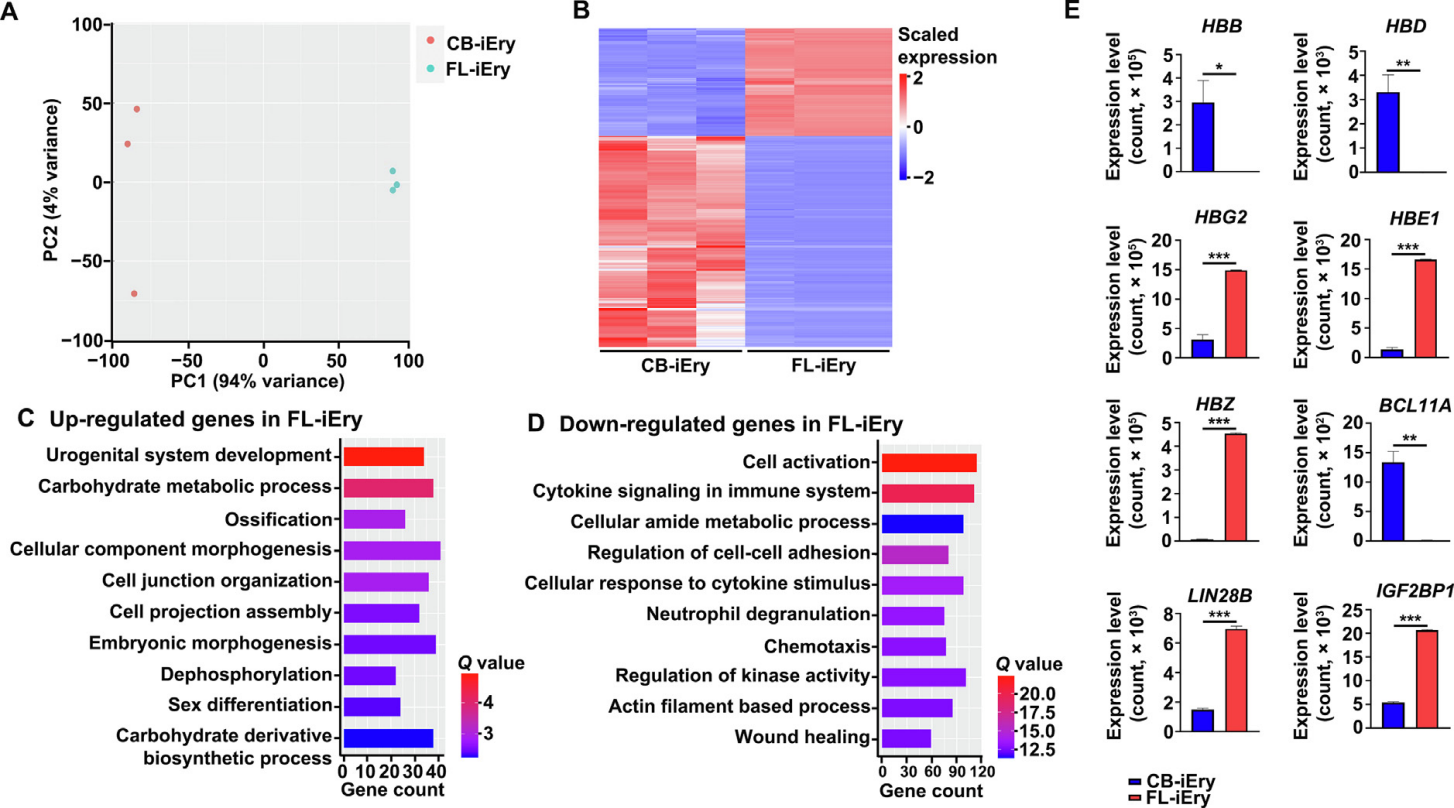
**Transcriptome comparison between FL and CB CD34^+^ cell-derived immortalized erythroid cells** **A.** PCA of immortalized cells generated from CB and FL CD34^+^ cells. **B.** Heatmap of expression of DEGs between CB-iEry and FL-iEry. **C.** Bar plot of enriched GO terms of up-regulated genes in FL-iEry. **D.** Bar plot of enriched GO terms of down-regulated genes in FL-iEry. **E.** Expression of hemoglobin genes and their regulators by normalized counts in CB-iEry and FL-iEry. The *Q* value represents the log-transformed adjusted *P* value.

Although FL-iEry and CB-iEry share the expression of some common characteristic genes, we were also interested in their differences. PCA showed a clear separation between FL-iEry and CB-iEry (Figure 9A). In total, 2488 DEGs were identified; the expression of 891 genes was up-regulated, and the expression of 1597 genes was down-regulated in FL-iEry (Table S8). The DEG expression pattern is represented by a heatmap in Figure 9B. GO analyses of the DEGs revealed enrichment of embryonic morphogenesis, reflecting the earlier stage of FL-iEry in development (Figure 9C). Genes with decreased expression levels in FL-iEry compared with CB-iEry were enriched in the regulation of cell activation and cytokine signaling in the immune system (Figure 9D), implying a possibly lower cytokine response in FL-iEry. Among all DEGs, we also examined the expression of different hemoglobin genes (Figure 9E). For β-like hemoglobin genes, the expression of the adult β hemoglobin genes *HBB* and *HBD* was significantly down-regulated, whereas the expression of the fetal γ hemoglobin gene *HBG2* and the embryonic ε hemoglobin gene *HBE1* was significantly up-regulated in FL-iEry. For α-like hemoglobin gene, the expression of the embryonic ζ hemoglobin gene *HBZ* was significantly up-regulated in FL-iEry. The increased expression levels of the embryonic hemoglobin genes *HBE1* and *HBZ* in FL-iEry compared with CB-iEry further indicate the earlier stage of FL-iEry than CB-iEry in development. Consistent with the up-regulated expression of γ hemoglobin in FL-iEry, the expression of *BCL11A*, which plays an important role in the γ globin to β globin switch [34], was down-regulated in FL-iEry compared with CB-iEry. In contrast, the expression levels of *LIN28B* and *IGF2BP1*, both of which promote γ globin expression [35], [36], were increased in FL-iEry compared with CB-iEry. These results indicate that FL-iEry and CB-iEry could be useful cellular models for studying the hemoglobin switch.

### Transcriptome comparison of various OrthoE proteins reveals common mechanisms for impaired enucleation

**Figure 10:**
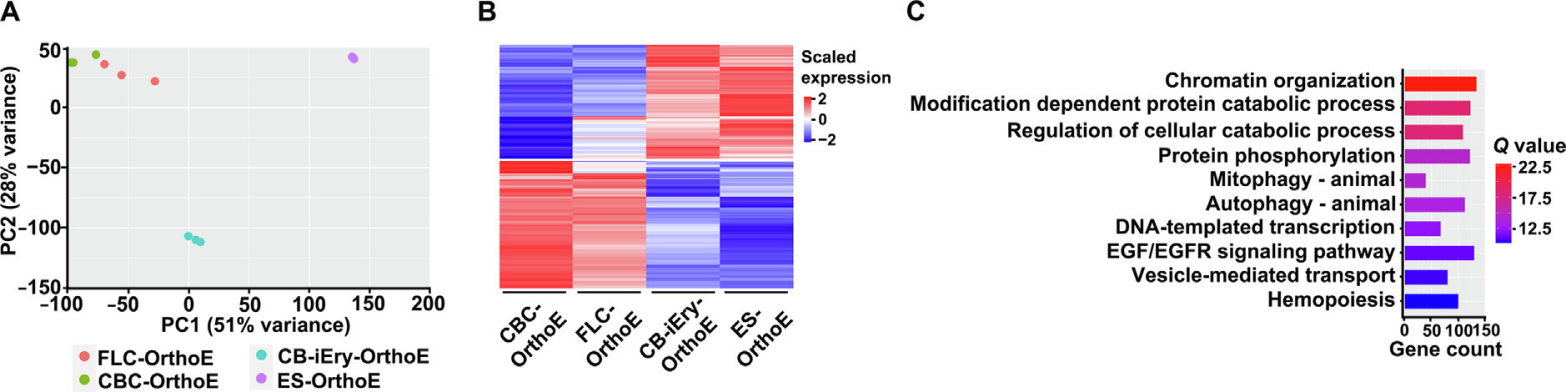
**Transcriptome comparison of OrthoEs from different sources** **A.** PCA of OrthoEs from four different sources. **B.** Heatmap of the expression of common DEGs between cells with and without enucleation capability. **C.** Bar plot of enriched GO terms of down-regulated genes in cells without enucleation capability. The *Q* value represents the log-transformed adjusted *P* value.

To investigate the mechanisms underlying the inability of OrthoEs derived from immortalized erythroid cell lines to enucleate, we performed RNA-seq on OrthoEs derived from CB-iEry (CB-iEry-OrthoEs) and compared the results with the transcriptomes of other OrthoEs, including FL CD34^+^ cell-derived OrthoEs (FLC-OrthoEs), CB CD34^+^ cell-derived OrthoEs (CB-OrthoEs) [17], and embryonic stem cell-derived OrthoEs (ES-OrthoEs) [13]. FLC-OrthoEs and CB-OrthoEs were able to enucleate, whereas CB-iEry-OrthoEs and ES-OrthoEs were not. PCA showed clear separation of the four cell groups as well as separation of cells with and without enucleation capability (Figure 10A). In total, 1929 DEGs were identified between these two groups (Figure 10B; Table S9). Genes with decreased expression in cells without enucleation capability were enriched in chromatin organization, protein phosphorylation, and mitophagy (Figure 10C). These GO enrichment terms are similar to the terms identified in OrthoEs derived from embryonic stem cells that also failed to enucleate [35]. We further examined the expression of genes known to affect enucleation, such as *HDAC5* [26], *FOXO3* [37], *XPO7* [38], *TRIM58* [39], *RIOK3* [40], and *TET3* [20]. The results showed that all of these genes were DEGs, and their expression was significantly down-regulated in OrthoEs without enucleation capability (Figure S5). Together, our findings strongly suggest that defects in chromatin organization, protein phosphorylation, and mitophagy are common mechanisms for the inability of immortalized OrthoEs and ES-OrthoEs to enucleate.

## Discussion

The FL is the key erythropoietic organ in the fetus. In contrast to extensive studies on mouse FL erythropoiesis [41], [42], very little is known about human FL erythropoiesis because of limitations in obtaining human FL tissue. In this study, we performed comprehensive characterization and stage-specific transcriptome analyses of human FL terminal erythropoiesis using primary human FL erythroblasts, *in vitro* cultured erythroblasts derived from human FL CD34^+^ cells, and a human FL CD34^+^ cell-derived immortalized erythroid cell line. Our study revealed novel aspects of human FL erythropoiesis. The transcriptomes we generated and the immortalized erythroid cell lines we established provide resources for future studies.

RNA-seq analyses of human erythroid cells have been performed using cultured erythroid cells *in vitro* from CB or PB CD34^+^ cells [17], [43]. Our study provides the first comprehensive analysis of the global transcriptome of primary human FL erythroblasts. The FL cells used in this study were obtained from 16 to 20 weeks of gestation. Given that same markers (Ter119, CD44) can be used to distinguish erythroblasts at different developmental stages from both adult bone and FL [9], [44], we speculate that these markers are unlikely to markedly change that much during fetal development. However, the transcriptomic profiles are likely to change. Unfortunately, the samples required to perform some tests were unavailable. Comparison of the transcriptomes between the primary human FL erythroblasts and the *in vitro* cultured erythroblasts derived from human FL CD34^+^ cells revealed several conserved features between *in vivo* and *in vitro* human FL terminal erythropoiesis. First, the numbers of expressed genes progressively decreased as terminal erythropoiesis proceeded. Decreasing numbers of expressed genes were also revealed in our previous RNA-seq analyses of erythroid cells derived from CB CD34^+^ cells [17], indicating a decrease in transcription activity during terminal erythropoiesis. Other commonly changed pathways include autophagy and chromatin modification, suggesting that these conserved biological processes are the core mechanisms for terminal erythropoiesis. In addition to conserved biological processes, we also found differences between *in vivo* and *in vitro* terminal erythropoiesis. First, the overall trajectory of *in vivo* and *in vitro* terminal erythroid differentiation was different. Whereas *in vivo* erythropoiesis showed a continuous differentiation process, *in vitro* erythropoiesis showed a two-part separation of the process (ProE/BasoE as one part and PolyE/OrthoE as the other part). Second, whereas the most significant changes in gene expression were detected between ProE and BasoE *in vivo*, the most significant changes were seen between BasoE and PolyE *in vitro*. Third, more DEGs were detected during *in vitro* than *in vivo* terminal erythropoiesis, particularly in the PolyE and OrthoE stages. One dramatic difference is that the pathway involved in lipid metabolism was significantly up-regulated in cultured erythroblasts at all stages. Together, our results provide strong suggestions regarding the environmental effects on gene expression during terminal erythropoiesis.

Immortalized erythroid cell lines have been established from CB, PB, and BM CD34^+^ cells [30], [31], [32]. However, the mechanisms and stages by which the cell lines are immortalized remain unclear. Our cytospin analyses, flow cytometry analyses of surface proteins, and global transcriptomic analyses of immortalized FL-iEry and CB-iEry demonstrated that the cells were immortalized at the ProE stage, indicating their usefulness for the study of terminal erythropoiesis but not for early-stage erythropoiesis. Moreover, the finding that the expression of telomere maintenance genes was up-regulated in FL-iEry and CB-iEry compared with nonimmortalized ProE suggests that the establishment of immortalized erythroid cell lines by the HPV-E6/E7 element occurs at least in part via the up-regulated expression of *TERT*. Another interesting finding of our study is that FL-iEry and CB-iEry expressed different types of hemoglobin and their regulators. The specific expression of hemoglobin *HBZ* in FL-iEry suggests the potential application of FL-iEry to studies of the α-like hemoglobin switch.

The enucleation rate of OrthoEs derived from ES cells, induced pluripotent stem cells, or immortalized erythroid cells is usually very low [13], [30], [45], [46]. However, the mechanisms underlying the impaired enucleation ability are largely unclear. We compared the transcriptomes of CB-iEry-derived OrthoEs, which failed to enucleate, with those of CB CD34^+^ cell-derived OrthoEs, which were able to enucleate [20], and found that expression of genes involved in chromatin organization and mitophagy was significantly down-regulated in OrthoEs without enucleation capability. Interestingly, down-regulation of chromatin organization and mitophagy was also detected in ES-derived erythroblasts that exhibited very low enucleation ability [13]. These findings indicate that defects in chromatin organization and mitophagy are key contributors to the inability of erythroblasts to enucleate. Strategies targeting these pathways should help improve enucleation.

## Materials and methods

### Antibodies

Mouse monoclonal antibodies against human band 3 and Kell were generated in our laboratory [3], [20], [21]. Commercial antibodies included BV421-conjugated CD45 (Catalog No. 563879, BD Biosciences, Franklin Lakes, NJ), PE-conjugated CD235a/GPA (Catalog No. 555570, BD Biosciences), APC-conjugated CD235a/GPA (Catalog No. 551336, BD Biosciences), PE-Cy7-conjugated CD123 (Catalog No. 25-1239-42, Invitrogen, Carlsbad, CA), APC-conjugated CD49d (Catalog No. 304307, BioLegend, San Diego, CA), PE-conjugated CD34 (Catalog No. 555822, BD Biosciences), APC-conjugated CD36 (Catalog No. 550956, BD Biosciences), FITC-conjugated CD36 (Catalog No. 555454, BD Biosciences), PE-conjugated CD49e (Catalog No. 103805, BioLegend), FITC-conjugated CD47 (Catalog No. 556045, BD Biosciences), PE-conjugated CD71 (Catalog No. 555537, BD Biosciences), AF647-conjugated CD147 (Catalog No. 562551, BD Biosciences), and Hoechst 33342 (Catalog No. 561908, BD Biosciences).

### Preparation of human FL single-cell suspension

Human FLs of gestational age ranging from 16 to 20 weeks were obtained from Advanced Bioscience Resources, Inc. (Alameda, CA). Single human FL cells were prepared as described previously [47]. Briefly, FL tissues were cut into small pieces and suspended in warm Hank’s solution plus 0.2% collagenase type IV (Catalog No. C5138, Sigma-Aldrich, St. Louis, MO), 2 U/ml DNase I (Catalog No. 4716728001, Sigma-Aldrich), 3.26 mM CaCl_2_ (Catalog No. 499609, Sigma-Aldrich), and 40 mM HEPES (Catalog No. 15630080, Gibco, Grand Island, NY). The tissues were incubated at 37 °C for 30 min. The cells were diluted with Iscove’s modified Dulbecco’s medium (IMDM; Catalog No. 12440053, Gibco) and passed through a 70-µm cell strainer. Centrifugation was performed at 100 *g* for 3 min. The supernatant was collected, and single cells were obtained.

### Flow cytometry analysis of human FL erythroblasts

Single human FL cells were suspended in staining buffer [phosphate-buffered saline (PBS) supplemented with 0.5% bovine serum albumin (BSA) and 2 mM ethylenediaminetetraacetic acid (EDTA)] at a concentration of 1 × 10^6^ and blocked with 0.4% human AB serum for 10 min. The cells were incubated with BV421-conjugated CD45, FITC-conjugated band 3, PE-conjugated GPA, and APC-conjugated α4-integrin at room temperature in the dark. After 15 min, the cells were washed with staining buffer by centrifugation at 300 *g* for 5 min. The supernatant was discarded, and the cell pellet was resuspended in staining buffer with 7-AAD (Catalog No. 559925, BD Biosciences). The cells were analyzed within 1 h of staining using a BD LSRFortessa cell analyzer (BD Biosciences).

### FACS of human FL erythroid precursors

Single human FL cells were separated by Ficoll density gradient centrifugation at 400 *g* for 30 min. The mononuclear cells were collected and incubated with CD45 magnetic microbeads (Catalog No. 130-045-801, Miltenyi Biotec, Bergisch Gladbach, Germany) for 30 min at 4 °C in the dark, according to the manufacturer’s protocol. The CD45^−^ cells were collected using a magnetic-activated cell sorting magnetic bead system. The isolated CD45^−^ cells were suspended in staining buffer (PBS supplemented with 2 mM EDTA and 0.5% BSA) and blocked with 0.4% human AB serum for 10 min. The cells were stained with PE-conjugated GPA, APC-conjugated α4-integrin, and FITC-conjugated band 3 at room temperature in the dark. After 15 min, the cells were washed with staining buffer by centrifugation at 300 *g* for 5 min at 4 °C and then resuspended in staining buffer with 7-AAD. The cells were finally sorted on a BD FACSAria fusion cell sorter (BD Biosciences).

### Purification and erythroid culture of human FL CD34^+^ cells

The FL single cells were separated on a Ficoll gradient as described above. CD34^+^ cells were purified from mononuclear cells using CD34 magnetic microbeads. The purified CD34^+^ cells were cultured in a three-phase liquid culture system [3], [20], [21]. In the first phase (day 0–day 7), CD34^+^ cells were cultured in IMDM with 3% human AB serum, 10 μg/ml insulin, 2% human plasma, 200 μg/ml holo-transferrin, 3 IU/ml heparin, 1 ng/ml IL-3, 3 IU/ml erythropoietin (EPO), 10 ng/ml stem cell factor (SCF), and 1% penicillin/streptomycin. In the second phase (day 7–day 11), the EPO concentration was decreased to 1 IU/ml, and the IL-3 was removed. In the third phase (day 11–day 17), the EPO concentration was decreased to 1 IU/ml, the holo-transferrin concentration was increased to 1 mg/ml, and the IL-3 and SCF were removed. The cells were cultured at 37 °C in 5% carbon dioxide.

### Monitoring of ***in vitro*** erythropoiesis of human FL CD34^+^ cells by flow cytometry analyses

For analysis of the early stage of erythropoiesis, 2 × 10^5^ cells in 25 μl staining buffer (PBS supplemented with 2 mM EDTA and 0.5% BSA) were stained with FITC-conjugated CD36, PE-conjugated CD34, APC-conjugated GPA, and PE-conjugated Cy7-IL3. To monitor terminal erythroid differentiation, 2 × 10^5^ cells in 25 μl staining buffer were stained with PE-conjugated GPA, APC-conjugated α4-integrin, and FITC-conjugated band 3. The stained cells were analyzed with the BD LSRFortessa system (BD Biosciences) as described above.

### Erythroid colony assay

Colony assays were performed as described previously [8], [21]. Briefly, the cells were diluted at a density of 200 cells by StemSpan serum-free expansion medium (SFEM; Vancouver, BC, Canada) in 1 ml of MethoCult H4434 for BFU-Es and MethoCult H4330 for CFU-Es, and they were then incubated at 37 °C in 5% carbon dioxide. CFU-E and BFU-E colonies were counted after 7 and 14 days of incubation, respectively.

### Construction of plasmids for immortalization

HPV-E6/E7 genomic DNA was subcloned and inserted into a pLVX-Tight-Puro backbone (a gift from Genmedic, China) downstream of the Tet operator by the restriction enzyme cutting sites *Eco*RI (NEB) and *Bam*HI (NEB). A tetracycline-controlled transcriptional activator pLVX-Tet-On Advanced plasmid was also prepared (a gift from Genmedic, China). The plasmids were sequenced by the Genewiz sequencing service (Azenta Life Sciences, Burlington, MA).

### Lentivirus preparation, transfection, and immortalization of FL CD34^+^ and CB CD34^+^ cells

Lentivirus was prepared as described in our previous studies [20], [21], [22]. For virus transfection, human CD34^+^ cells isolated from FL or CB were cultured in differentiation medium (phase 1 medium of three-phase medium, described above) for 2 days. On day 2, the cells were transduced with the lentiviral vector pLVX-Tight-Puro-E6/E7, as described previously. On day 3, the medium was changed to fresh medium. On day 4, transduced cells were selected by puromycin (1 μg/ml) and G418 (400 μg/ml). On day 5, the cells were transferred into expansion medium (SFEM containing 50 ng/ml SCF, 3 IU/ml EPO, 1 μM dexamethasone, and 1 μg/ml doxycycline). The cells were then cultured in the expansion medium for more than 60 days to select the immortalized cells. The medium was changed to fresh medium every other day, and the cell density was maintained at < 2 × 10^5^ cells/ml. When the growth curve became stable, single cells were sorted into a 96-well plate to select the immortalized cell colonies.

### Terminal differentiation of immortalized erythroid cell lines

For immortalized cell differentiation, the cells were transferred into differentiation medium for 6 days along with doxycycline. After day 6, the cells were cultured in IMDM containing 3% AB serum, 2% PB plasma, 10 μg/ml insulin, 3 IU/ml heparin, 3 IU/ml EPO, 1 mg/ml transferrin, and 1% penicillin/streptomycin.

### CRISPR/Cas9-mediated gene knockout in immortalized cell lines

Single guide RNAs (sgRNAs) targeting *CD44* (forward: 5′-CACCGCGTGGAATACACCTGCAAAG-3′, reverse: 5′-AAACCTTTGCAGGTGTATTCCACGC-3′) or *CD147* (forward: 5′-CACCGCGTCAGAACACATCAACGAG-3′, reverse: 5′-AAACCTCGTTGATGTGTTCTGACGC-3′) were designed by CRISPR design tools (http://crispr.mit.edu/). sgRNAs were synthesized by Eurofins Scientific (Luxembourg, UK). sgRNAs were constructed into a CRISPR-Cas9-V3 vector (kindly provided by Xianfang Wu at Rockefeller University) at the *Bbs*I restriction site. Lentivirus packaging and virus transduction procedures were performed as described previously [21], [22]. On day 6 posttransduction, GFP^+^CD44^−^ or GFP^+^BSG^−^ single cells were sorted into 96-well plates. After culturing for 2 weeks, clones were picked for validation of *CD44* or *BSG* gene knockout by genome DNA sequencing.

### Cytospin and May–Grünwald–Giemsa staining

Cytospins were prepared on coated slides with 1 × 10^5^ cells using the Thermo Fisher Scientific Shandon Cytospin 4 centrifuge. The slides were then stained with May–Grünwald–Giemsa solution (Catalog No. MG500, Sigma Aldrich) for 5 min. After rinsing for 90 s in 40 mM Tris buffer at pH 7.4, the slides were stained with Giemsa solution (Catalog No. GS500, Sigma-Aldrich) for 15 min. Finally, the slides were rinsed twice with water. The cells were imaged using a Leica DM2000 inverted microscope.

### RNA-seq and analysis

We used a QIAGEN RNA isolation kit (Catalog No. 74104, QIAGEN, Dusseldorf, Germany) to extract RNA. Samples with an RNA integrity number > 9 were used for construction of a cDNA library by an Illumina TruSeq kit (Illumina, San Diego, CA). The sequencing strategy was 100 bp paired-end by an Illumina HiSeq 4000. We used the RNA-seq data of CB-derived erythroblasts from a previous study [29]. Low-quality reads were removed and filtered by fastp. Filtered reads were quantified by Kallisto using the hg19 transcriptome index [48]. Pairwise comparison of two groups was performed by DESeq2 [49], and the batch effect was included in the design of DESeq2. The cutoffs of fold change ≥ 2, adjusted *P* ≤ 0.05, and ≥ 1 transcript per million in at least one group were adopted to identify DEGs in the pairwise comparisons. Log-transformed normalized counts were used in PCA. GO enrichment analyses were applied by Metascape [50]. GO terms with a *Q* value < 0.001 were considered significant, and the top 10 terms with the smallest *Q* values are listed in the results. For transcriptome analysis of primary or cultured terminal erythropoiesis, expression patterns were analyzed on all pairwise adjacent DEGs by the time-course clustering R package TCseq using log-transformed normalized counts. For comparison between primary and cultured terminal erythropoiesis, stage-specific DEGs between the same stage of primary and cultured erythroblasts were identified and clustered by TCseq. For transcriptome analysis, we only used late BasoE as BasoE in the comparison.

## Ethical statement

All experimental procedures involving human cells were approved by the Ethics Committee of Zhengzhou University, China (Approval No. ZZUIRB 2023-184).

## Data availability

The datasets generated in the current study have been deposited in the Genome Sequence Archive for Human [51] at the National Genomics Data Center, Beijing Institute of Genomics, Chinese Academy of Sciences / China National Center for Bioinformatics (GSA-Human: HRA004488), and are publicly accessible at https://ngdc.cncb.ac.cn/gsa-human/.

## Competing interests

The authors have declared no competing interests.

## CRediT authorship contribution statement

**Yongshuai Han:** Conceptualization, Investigation, Resources, Writing – original draft. **Shihui Wang:** Conceptualization, Formal analysis, Investigation, Visualization, Writing – original draft. **Yaomei Wang:** Conceptualization, Investigation, Resources. **Yumin Huang:** Conceptualization, Investigation, Resources. **Chengjie Gao:** Conceptualization, Resources. **Xinhua Guo:** Conceptualization, Resources. **Lixiang Chen:** Writing – review & editing. **Huizhi Zhao:** Conceptualization, Software, Formal analysis, Visualization, Writing – original draft. **Xiuli An:** Conceptualization, Investigation, Writing – original draft, Writing – review & editing. All authors have read and approved the final manuscript.

## Supplementary material

Supplementary Figure S1 **GPA, band 3, and α4-integrin enable the isolation of erythroblasts from human fetal liver A.** Flow cytometry analysis of primary erythroblasts of human fetal liver using GPA, band3, and α4-integrin. **B.** Representative images of sorted erythroblasts from human fetal liver. Scale bar = 10 µm. GPA

Supplementary Figure S2 **PCA of terminal erythroblasts from human fetal liver *in vivo* and *in vitro***

Supplementary Figure S3 **Gene expression and GO enrichment of nine clusters of all differentially expressed genes from the same-stage comparison between human fetal liver *in vivo* and *in vitro*** A heatmap of the gene expression of all differentially expressed genes in the nine clusters is shown in the left panel. Curve representations of different expression levels of genes in nine clusters are shown in the middle panel. Bar plots of enriched GO terms of genes in nine clusters are shown in the right panel. The *Q* value represents the log-transformed adjusted *P*

Supplementary Figure S4 **RNA-seq analyses of immortalized erythroid cells****A.** Principal component analyses of immortalized erythroid cells and each stage of terminal erythroblasts cultured from cord blood and fetal liver CD34+ cells. **B****.** Heatmap of common differentially expressed genes of two separate comparisons between immortalized erythroid cells and their counterpart ProE from the same source. **C****.** Bar plot of enriched GO terms of upregulated genes in immortalized cells. **D****.** Bar plot of TERT expression in ProEs and immortalized erythroblasts by normalized counts. The *Q* value represents the log-transformed adjusted *P*

Supplementary Figure S5 **Gene expression comparison of known enucleation-related genes in cultured OrthoEs** Gene expression levels are represented by log2 normalized counts. CBC-OrthoE, cord blood CD34+ cell-derived OrthoE; FLC-OrthoE, fetal liver CD34+ cell-derived OrthoE; CB-iEry-OrthoE, cord blood immortalized erythroid cell-derived OrthoE; ES-OrthoE, embryonic stem cell-derived OrthoE. ***, adjusted *P* < 0.0001

Supplementary Table S1 **Expressed genes in FL *in vivo* terminal erythropoiesis**

Supplementary Table S2 **DEGs in FL *in vivo* terminal erythropoiesis**

Supplementary Table S3 **Expressed genes in FL *in vitro* terminal erythropoiesis**

Supplementary Table S4 **DEGs in FL *in vitro* terminal erythropoiesis**

Supplementary Table S5 **Stage-specific genes in FL *in vitro* and *in vivo* terminal erythropoiesis**

Supplementary Table S6 **DEGs of same-stage comparison between FL *in vitro* and *in vivo* terminal erythropoiesis**

Supplementary Table S7 **DEGs between immortalized erythroblast and cultured ProE**

Supplementary Table S8 **DEGs between CB-iEry and FL-iEry**

Supplementary Table S9 **DEGs between OrthoEs with and without enucleation capability**

## References

[1] Palis J., Robertson S., Kennedy M., Wall C., Keller G. (1999). Development of erythroid and myeloid progenitors in the yolk sac and embryo proper of the mouse. Development.

[2] Chen L., Wang J., Liu J., Wang H., Hillyer C.D., Blanc L. (2021). Dynamic changes in murine erythropoiesis from birth to adulthood: implications for the study of murine models of anemia. Blood Adv.

[3] Hu J., Liu J., Xue F., Halverson G., Reid M., Guo A. (2013). Isolation and functional characterization of human erythroblasts at distinct stages: implications for understanding of normal and disordered erythropoiesis *in vivo*. Blood.

[4] Nandakumar S.K., Ulirsch J.C., Sankaran V.G. (2016). Advances in understanding erythropoiesis: evolving perspectives. Br J Haematol.

[5] Menon V., Ghaffari S. (2021). Erythroid enucleation: a gateway into a “bloody” world. Exp Hematol.

[6] Flygare J., Rayon Estrada V., Shin C., Gupta S., Lodish H.F. (2011). HIF1α synergizes with glucocorticoids to promote BFU-E progenitor self-renewal. Blood.

[7] Liu J., Zhang J., Ginzburg Y., Li H., Xue F., De Franceschi L. (2013). Quantitative analysis of murine terminal erythroid differentiation *in vivo*: novel method to study normal and disordered erythropoiesis. Blood.

[8] Li J., Hale J., Bhagia P., Xue F., Chen L., Jaffray J. (2014). Isolation and transcriptome analyses of human erythroid progenitors: BFU-E and CFU-E. Blood.

[9] Zhang H., Wang S., Liu D., Gao C., Han Y., Guo X. (2021). EpoR-tdTomato-Cre mice enable identification of EpoR expression in subsets of tissue macrophages and hematopoietic cells. Blood.

[10] Yan H., Ali A., Blanc L., Narla A., Lane J.M., Gao E. (2021). Comprehensive phenotyping of erythropoiesis in human bone marrow: evaluation of normal and ineffective erythropoiesis. Am J Hematol.

[11] Caulier A.L., Sankaran V.G. (2022). Molecular and cellular mechanisms that regulate human erythropoiesis. Blood.

[12] Xu C., He J., Wang H., Zhang Y., Wu J., Zhao L. (2022). Single-cell transcriptomic analysis identifies an immune-prone population in erythroid precursors during human ontogenesis. Nat Immunol.

[13] Wang S., Zhao H., Zhang H., Gao C., Guo X., Chen L. (2022). Analyses of erythropoiesis from embryonic stem cell-CD34^+^ and cord blood-CD34^+^ cells reveal mechanisms for defective expansion and enucleation of embryomic stem cell-erythroid cells. J Cell Mol Med.

[14] Dulmovits B.M., Tang Y., Papoin J., He M., Li J., Yang H. (2022). HMGB1-mediated restriction of EPO signaling contributes to anemia of inflammation. Blood.

[15] Wang B., Wang C., Wan Y., Gao J., Ma Y., Zhang Y. (2022). Decoding the pathogenesis of Diamond-Blackfan anemia using single-cell RNA-seq. Cell Discov.

[16] Yu L., Lemay P., Ludlow A., Guyot M.C., Jones M., Mohamed F.F. (2021). A new murine *Rpl5* (*uL18*) mutation provides a unique model of variably penetrant Diamond-Blackfan anemia. Blood Adv.

[17] An X., Schulz V.P., Li J., Wu K., Liu J., Xue F. (2014). Global transcriptome analyses of human and murine terminal erythroid differentiation. Blood.

[18] Gautier E.F., Ducamp S., Leduc M., Salnot V., Guillonneau F., Dussiot M. (2016). Comprehensive proteomic analysis of human erythropoiesis. Cell Rep.

[19] Schulz V.P., Yan H., Lezon-Geyda K., An X., Hale J., Hillyer C.D. (2019). A unique epigenomic landscape defines human erythropoiesis. Cell Rep.

[20] Yan H., Wang Y., Qu X., Li J., Hale J., Huang Y. (2017). Distinct roles for TET family proteins in regulating human erythropoiesis. Blood.

[21] Qu X., Zhang S., Wang S., Wang Y., Li W., Huang Y. (2018). TET2 deficiency leads to stem cell factor-dependent clonal expansion of dysfunctional erythroid progenitors. Blood.

[22] Han X., Zhang J., Peng Y., Peng M., Chen X., Chen H. (2017). Unexpected role for p19^INK4d^ in posttranscriptional regulation of GATA1 and modulation of human terminal erythropoiesis. Blood.

[23] Giarratana M.C., Kobari L., Lapillonne H., Chalmers D., Kiger L., Cynober T. (2005). *Ex vivo* generation of fully mature human red blood cells from hematopoietic stem cells. Nat Biotechnol.

[24] Neildez-Nguyen T.M., Wajcman H., Marden M.C., Bensidhoum M., Moncollin V., Giarratana M.C. (2002). Human erythroid cells produced *ex vivo* at large scale differentiate into red blood cells *in vivo*. Nat Biotechnol.

[25] Jin H., Kim H.S., Kim S., Kim H.O. (2014). Erythropoietic potential of CD34+ hematopoietic stem cells from human cord blood and G-CSF-mobilized peripheral blood. Biomed Res Int.

[26] Wang Y., Li W., Schulz V.P., Zhao H., Qu X., Qi Q. (2021). Impairment of human terminal erythroid differentiation by histone deacetylase 5 deficiency. Blood.

[27] Lee E., Sivalingam J., Lim Z.R., Chia G., Shi L.G., Roberts M. (2018). Review: *in vitro* generation of red blood cells for transfusion medicine: progress, prospects and challenges. Biotechnol Adv.

[28] Gibson J.S., Rees D.C. (2018). Lipid metabolism in terminal erythropoiesis. Blood.

[29] Huang N.J., Lin Y.C., Lin C.Y., Pishesha N., Lewis C.A., Freinkman E. (2018). Enhanced phosphocholine metabolism is essential for terminal erythropoiesis. Blood.

[30] Kurita R., Suda N., Sudo K., Miharada K., Hiroyama T., Miyoshi H. (2013). Establishment of immortalized human erythroid progenitor cell lines able to produce enucleated red blood cells. PLoS One.

[31] Trakarnsanga K., Griffiths R.E., Wilson M.C., Blair A., Satchwell T.J., Meinders M. (2017). An immortalized adult human erythroid line facilitates sustainable and scalable generation of functional red cells. Nat Commun.

[32] Daniels D.E., Ferguson D.C.J., Griffiths R.E., Trakarnsanga K., Cogan N., MacInnes K.A. (2021). Reproducible immortalization of erythroblasts from multiple stem cell sources provides approach for sustainable RBC therapeutics. Mol Ther Methods Clin Dev.

[33] Sealey D.C., Zheng L., Taboski M.A., Cruickshank J., Ikura M., Harrington L.A. (2010). The N-terminus of hTERT contains a DNA-binding domain and is required for telomerase activity and cellular immortalization. Nucleic Acids Res.

[34] Liu N., Hargreaves V.V., Zhu Q., Kurland J.V., Hong J., Kim W. (2018). Direct promoter repression by BCL11A controls the fetal to adult hemoglobin switch. Cell.

[35] Basak A., Munschauer M., Lareau C.A., Montbleau K.E., Ulirsch J.C., Hartigan C.R. (2020). Control of human hemoglobin switching by LIN28B-mediated regulation of *BCL11A* translation. Nat Genet.

[36] Chambers C.B., Gross J., Pratt K., Guo X., Byrnes C., Lee Y.T. (2020). The mRNA-binding protein IGF2BP1 restores fetal hemoglobin in cultured erythroid cells from patients with 1 hemoglobin disorders. Mol Ther Methods Clin Dev.

[37] Liang R., Campreciós G., Kou Y., McGrath K., Nowak R., Catherman S. (2015). A systems approach identifies essential FOXO3 functions at key steps of terminal erythropoiesis. PLoS Genet.

[38] Figueroa A.A., Fasano J.D., Martinez-Morilla S., Venkatesan S., Kupfer G., Hattangadi S.M. (2018). miR-181a regulates erythroid enucleation via the regulation of Xpo7 expression. Haematologica.

[39] Thom C.S., Traxler E.A., Khandros E., Nickas J.M., Zhou O.Y., Lazarus J.E. (2014). Trim58 degrades Dynein and regulates terminal erythropoiesis. Dev Cell.

[40] Zhang L., Flygare J., Wong P., Lim B., Lodish H.F. (2011). miR-191 regulates mouse erythroblast enucleation by down-regulating *Riok3* and *Mxi1*. Genes Dev.

[41] Isern J., Fraser S.T., He Z., Baron M.H. (2008). The fetal liver is a niche for maturation of primitive erythroid cells. Proc Natl Acad Sci U S A.

[42] Fantin A., Tacconi C., Villa E., Ceccacci E., Denti L., Ruhrberg C. (2021). KIT is required for fetal liver hematopoiesis. Front Cell Dev Biol.

[43] Yan H., Hale J., Jaffray J., Li J., Wang Y., Huang Y. (2018). Developmental differences between neonatal and adult human erythropoiesis. Am J Hematol.

[44] Chen K., Liu J., Heck S., Chasis J.A., An X., Mohandas N. (2009). Resolving the distinct stages in erythroid differentiation based on dynamic changes in membrane protein expression during erythropoiesis. Proc Natl Acad Sci U S A.

[45] Olivier E.N., Qiu C., Velho M., Hirsch R.E., Bouhassira E.E. (2006). Large-scale production of embryonic red blood cells from human embryonic stem cells. Exp Hematol.

[46] Qiu C., Olivier E.N., Velho M., Bouhassira E.E. (2008). Globin switches in yolk sac-like primitive and fetal-like definitive red blood cells produced from human embryonic stem cells. Blood.

[47] Pourcher G., Mazurier C., King Y.Y., Giarratana M.C., Kobari L., Boehm D. (2011). Human fetal liver: an *in vitro* model of erythropoiesis. Stem Cells Int.

[48] Bray N.L., Pimentel H., Melsted P., Pachter L. (2016). Near-optimal probabilistic RNA-seq quantification. Nat Biotechnol.

[49] Love M.I., Huber W., Anders S. (2014). Moderated estimation of fold change and dispersion for RNA-seq data with DESeq2. Genome Biol.

[50] Zhou Y., Zhou B., Pache L., Chang M., Khodabakhshi A.H., Tanaseichuk O. (2019). Metascape provides a biologist-oriented resource for the analysis of systems-level datasets. Nat Commun.

[51] Chen T., Chen X., Zhang S., Zhu J., Tang B., Wang A. (2021). The Genome Sequence Archive Family: toward explosive data growth and diverse data types. Genomics Proteomics Bioinformatics.

