# Supplementary figures and images for: Comprehensive Characterization and Global Transcriptome Analysis of Human Fetal Liver Terminal Erythropoiesis
Source: Genomics Proteomics Bioinformatics. 2023 Aug 30;21(6):1117–32. doi: 10.1016/j.gpb.2023.07.001 (PMC11082260; doi:10.1016/j.gpb.2023.07.001)

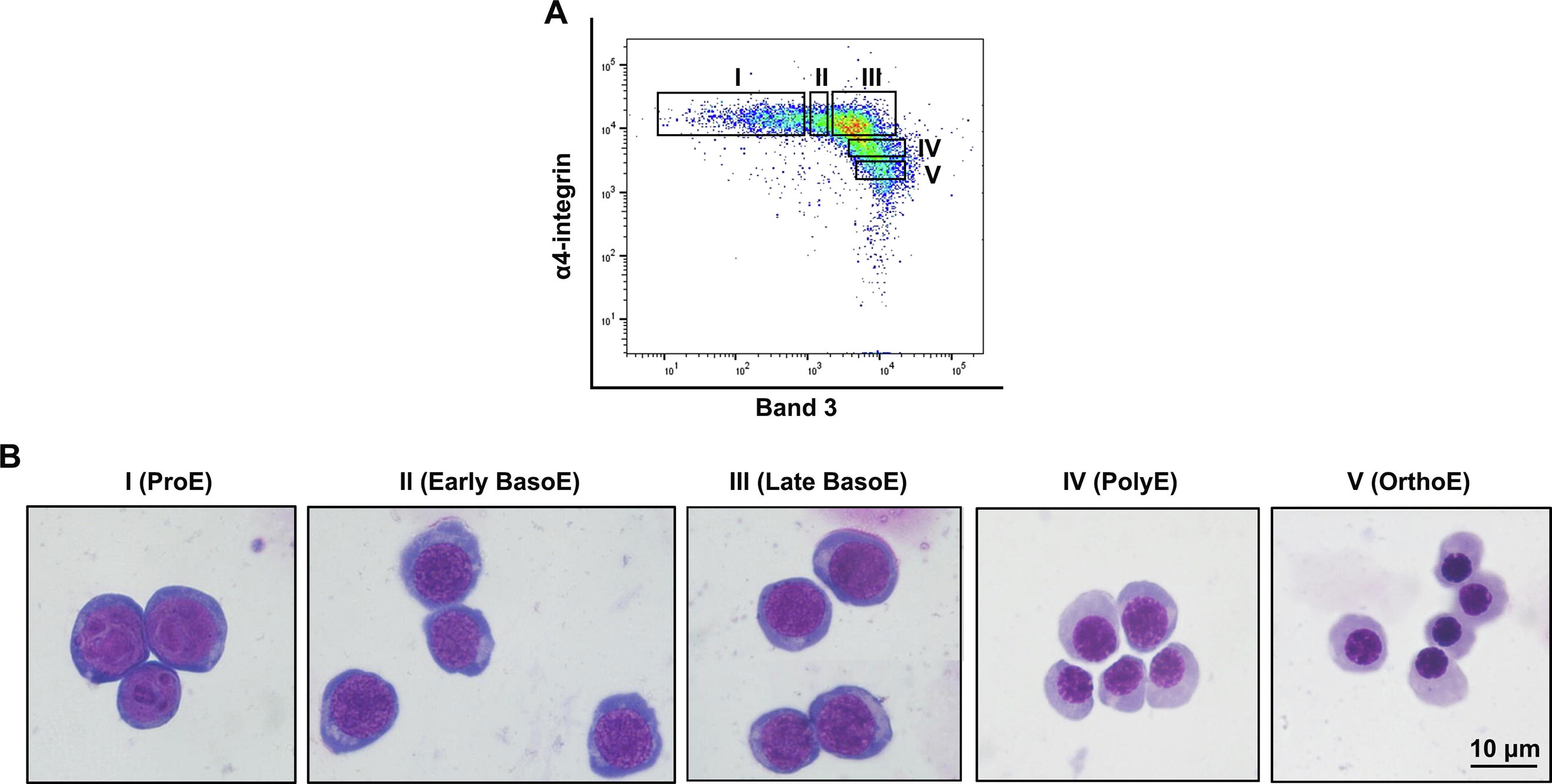

Supplement: Supplementary Figure S1 — GPA, band 3, and α4-integrin enable the isolation of erythroblasts from human fetal liver A. Flow cytometry analysis of primary erythroblasts of human fetal liver using GPA, band3, and α4-integrin. B. Representative images of sorted erythroblasts from human fetal liver. Scale bar = 10 µm. GPA [file mmc1.jpg]

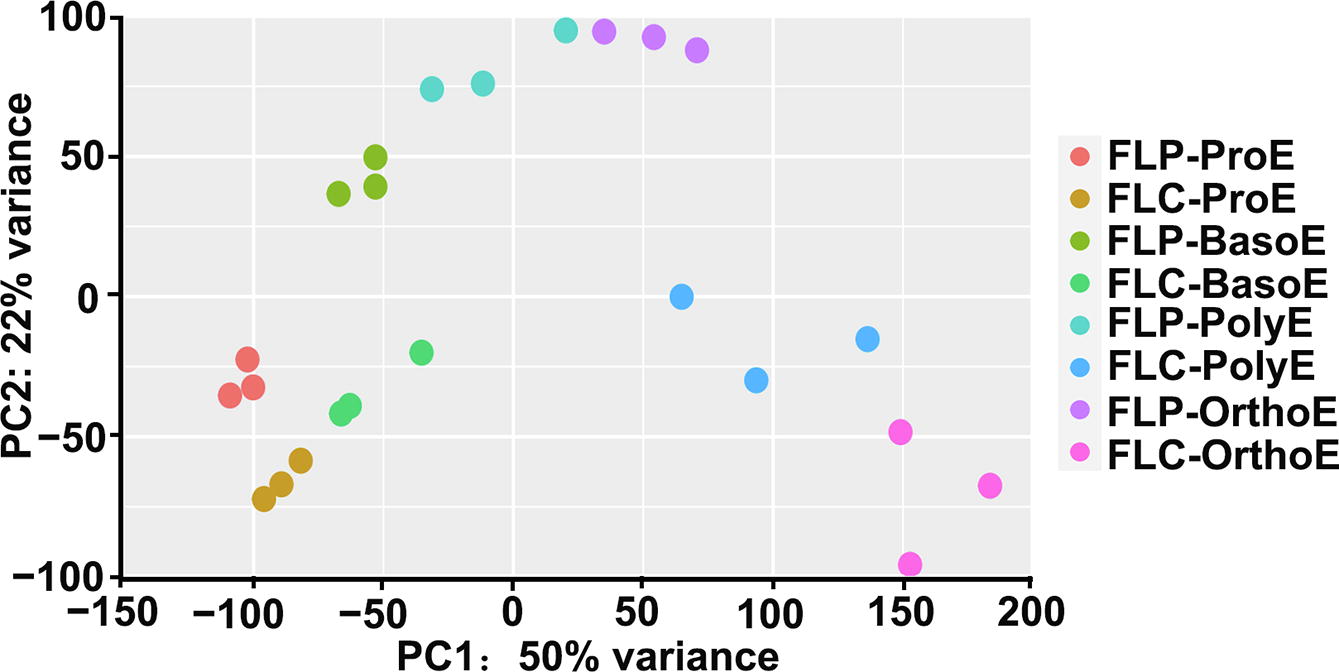

Supplement: Supplementary Figure S2 — PCA of terminal erythroblasts from human fetal liver in vivo and in vitro [file mmc2.jpg]

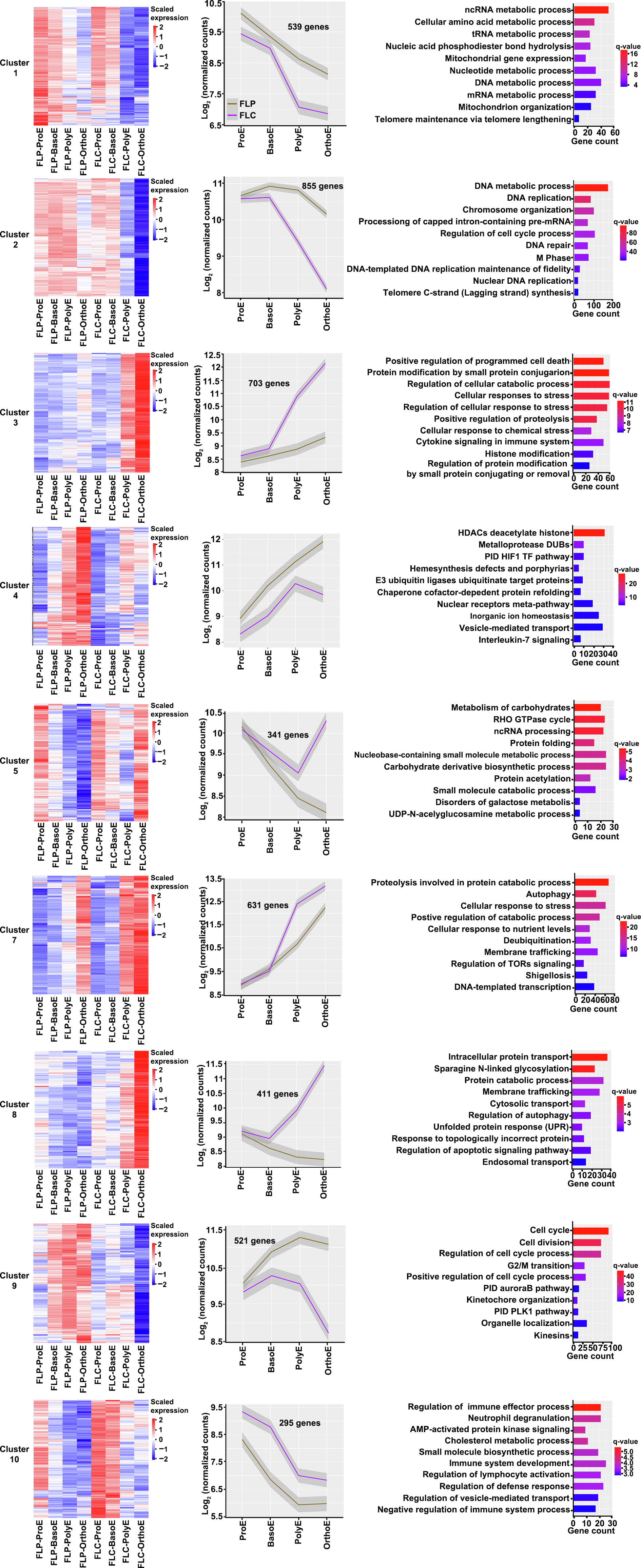

Supplement: Supplementary Figure S3 — Gene expression and GO enrichment of nine clusters of all differentially expressed genes from the same-stage comparison between human fetal liver in vivo and in vitro A heatmap of the gene expression of all differentially expressed genes in the nine clusters is shown in the left panel. Curve representations of different expression levels of genes in nine clusters are shown in the middle panel. Bar plots of enriched GO terms of genes in nine clusters are shown in the right panel. The Q value represents the log-transformed adjusted P [file mmc3.jpg]

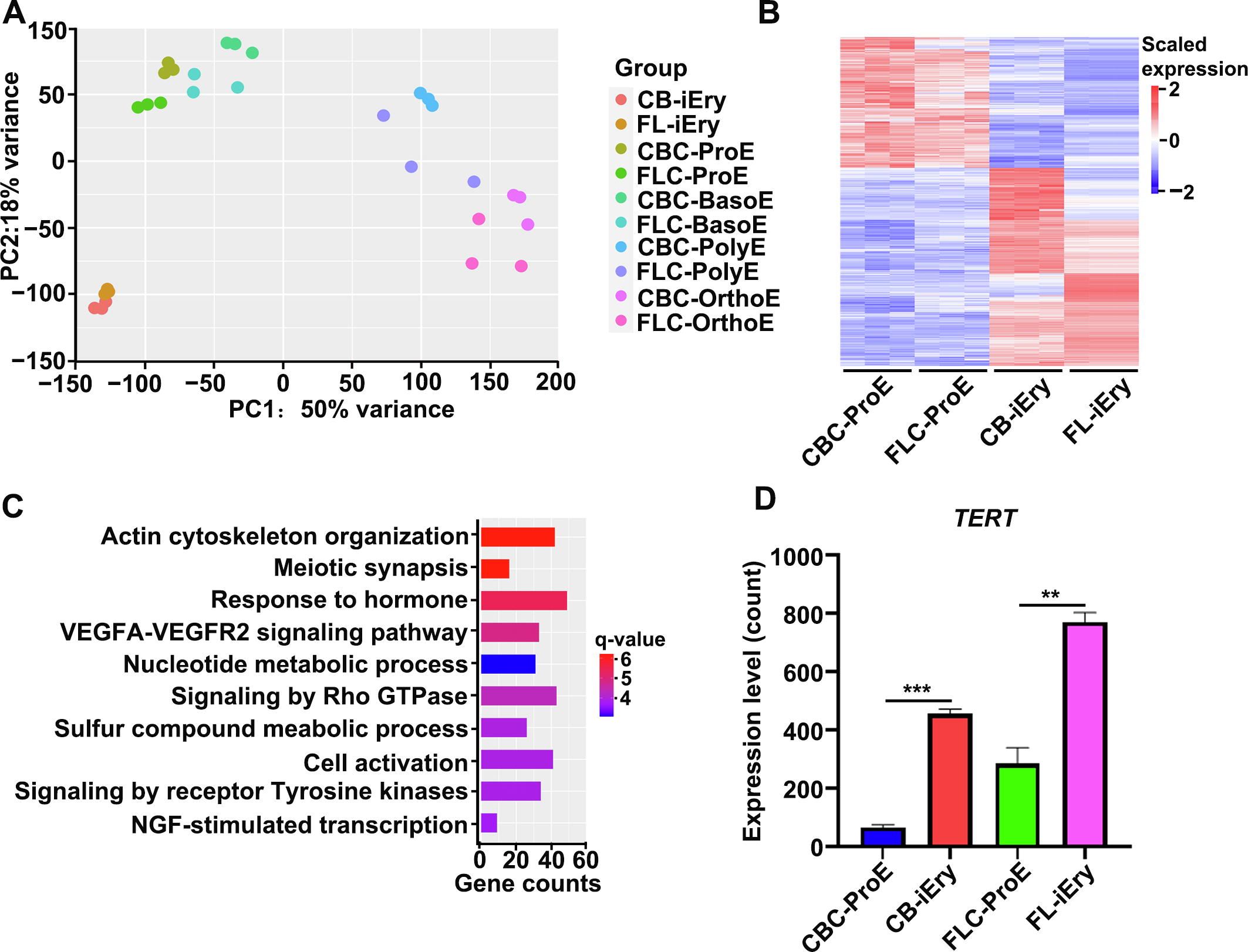

Supplement: Supplementary Figure S4 — RNA-seq analyses of immortalized erythroid cellsA. Principal component analyses of immortalized erythroid cells and each stage of terminal erythroblasts cultured from cord blood and fetal liver CD34+ cells. B. Heatmap of common differentially expressed genes of two separate comparisons between immortalized erythroid cells and their counterpart ProE from the same source. C. Bar plot of enriched GO terms of upregulated genes in immortalized cells. D. Bar plot of TERT expression in ProEs and immortalized erythroblasts by normalized counts. The Q value represents the log-transformed adjusted P [file mmc4.jpg]

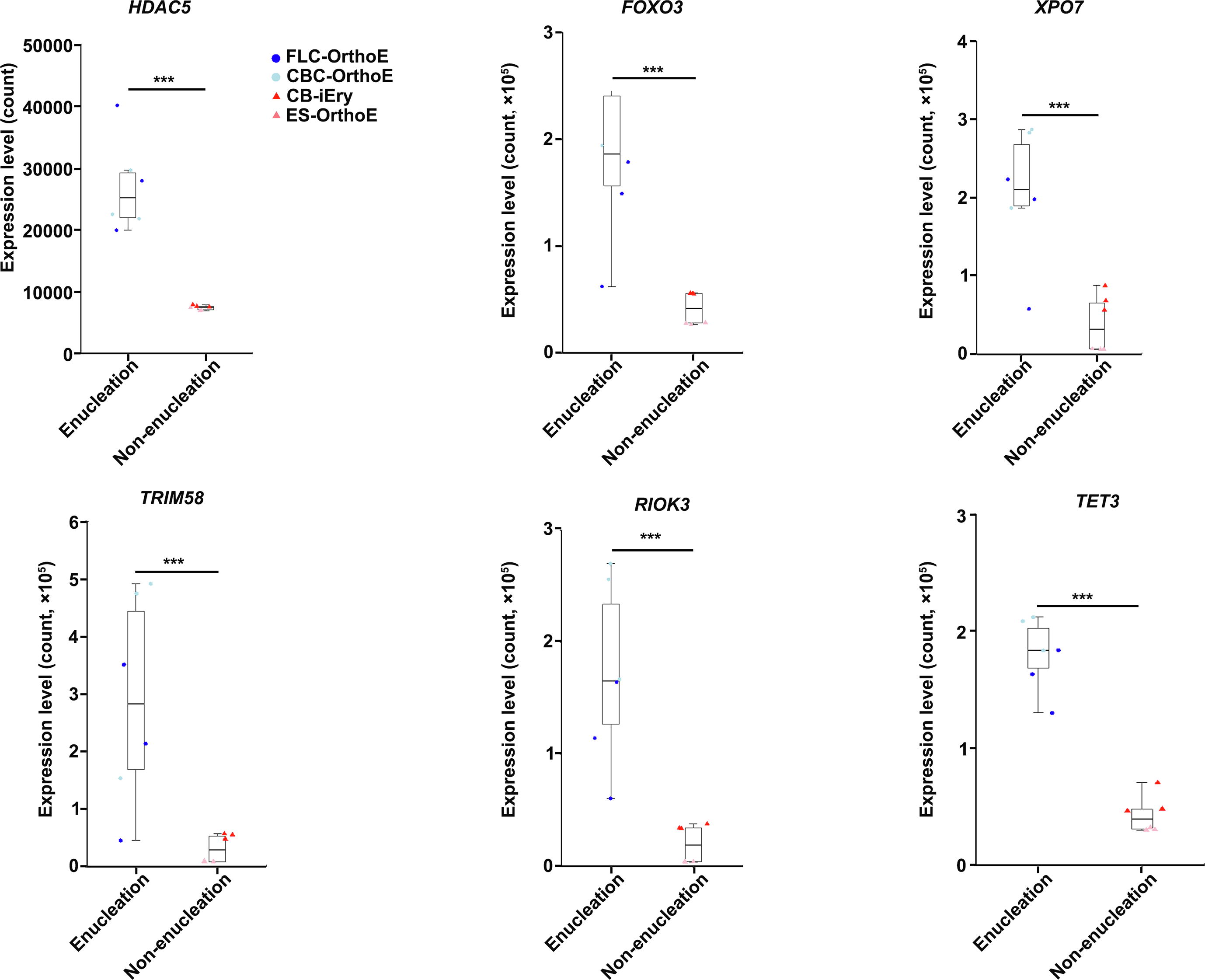

Supplement: Supplementary Figure S5 — Gene expression comparison of known enucleation-related genes in cultured OrthoEs Gene expression levels are represented by log2 normalized counts. CBC-OrthoE, cord blood CD34+ cell-derived OrthoE; FLC-OrthoE, fetal liver CD34+ cell-derived OrthoE; CB-iEry-OrthoE, cord blood immortalized erythroid cell-derived OrthoE; ES-OrthoE, embryonic stem cell-derived OrthoE. ***, adjusted P < 0.0001 [file mmc5.jpg]
